# Dissolved organic matter specialization drives temporal dynamics of simplified bacterial communities in a microcosm experiment

**DOI:** 10.1093/ismeco/ycag045

**Published:** 2026-02-28

**Authors:** Sarah R Sandor, Thomas Scheuerl, Jeremy A Fonvielle, Caroline Kemp, Andrew J Tanentzap

**Affiliations:** Ecosystems and Global Change Group, Department of Plant Sciences, University of Cambridge, Cambridge, CB2 3EA, United Kingdom; Institute for Chemistry and Biology of the Marine Environment, University of Oldenburg, 26129 Oldenburg, Germany; Ecosystems and Global Change Group, Department of Plant Sciences, University of Cambridge, Cambridge, CB2 3EA, United Kingdom; Research Department for Limnology, University of Innsbruck, 5310 Mondsee, Austria; Ecosystems and Global Change Group, Department of Plant Sciences, University of Cambridge, Cambridge, CB2 3EA, United Kingdom; Ecosystems and Global Change Group, Department of Plant Sciences, University of Cambridge, Cambridge, CB2 3EA, United Kingdom; Ecosystems and Global Change Group, Department of Plant Sciences, University of Cambridge, Cambridge, CB2 3EA, United Kingdom; Institute for Chemistry and Biology of the Marine Environment, University of Oldenburg, 26129 Oldenburg, Germany; Ecosystems and Global Change Group, School of Environment, Trent University, Peterborough, ON, K9L 0G2, Canada

**Keywords:** microbial community succession, dissolved organic matter degradation, microbe–DOM interactions, climate change

## Abstract

Microorganisms form the base of aquatic food webs and play a key role in the global carbon cycle by decomposing dissolved organic matter (DOM). Climate change is predicted to shift the composition of DOM in northern freshwaters from predominantly small, low molecular weight compounds to aromatic, high molecular weight compounds. However, the consequences of these changes for bacterial communities and their role in wider ecosystem processes are poorly understood. Here, we used a 14-day incubation experiment to test how the same bacterial community responded to diverse DOM sources that varied in their bioavailability and were representative of predicted compositional changes in northern waters. Using full-length 16S amplicon sequencing, we found that bacterial communities differed in their composition across sources within 24 h of exposure to novel DOM, but changed similarly over time thereafter, primarily driven by consistent increases in the relative abundance of one generalist species. Microbial reworking of DOM, characterized using ultra-high-resolution mass spectrometry, led to an increase in the relative abundance of less bioavailable compounds on sources with higher initial bioavailability. Our study advances previous work by suggesting that interactions between bacteria and DOM under novel environmental conditions depend on their level of specialization and that any losses in resource specialization may have consequences for the persistence and trophic transfer of carbon in aquatic food webs.

## Introduction

By decomposing and producing dissolved organic matter (DOM), aquatic microbes regulate global biogeochemical cycles and mobilize energy and nutrients into food webs [1, 2]. Climate change is altering DOM composition in ways that may push microorganisms beyond their ecological niches. DOM comprises thousands of unique carbon-based molecules that vary in their reactivity and bioavailability for microbial degradation [3, 4]. These molecules range from small biomolecules, including carbohydrates, amino acids, and proteins, to larger partially degraded plant material like lignin and humic acids [5]. Low molecular weight molecules, such as from algal or microbial exudates, are typically mineralized by bacteria into CO_2_ because they are more bioavailable [6, 7], i.e. yield greater energetic returns upon oxidation. By contrast, terrestrial-derived plant molecules, such as lignin, are typically less bioavailable because they yield less energy upon oxidation [6, 8]. These compounds therefore tend to be incorporated into bacterial biomass, transferring carbon to higher trophic levels [1, 6, 8, 9], though they may offset any food web subsidy by shading primary production [10]. Northern freshwaters are specifically predicted to shift in DOM composition in coming decades due to climate change from small, aliphatic compounds to higher molecular weight, aromatic, and humic-like compounds derived from terrestrial landscapes [11]. Any changes in the bioavailability of DOM may therefore have large ecological impacts in freshwaters, such as on nutrient availability and transfer efficiency through food webs [8, 11].

Because different bacterial species degrade different compounds [12–15], the extent to which bacteria are specialized to degrade these substrates is a major factor determining their response and adaptation to novel conditions [16]. Bacteria specialized on specific resources, for instance, labile, low molecular weight DOM, typically dominate communities when the resource is abundant, but disappear in its absence [13, 15, 17]. By contrast, generalist species can maintain their population sizes even with changes in DOM composition because they grow across a broader range of environmental conditions [13]. Therefore, generalists may persist in communities as climate change shifts DOM composition in northern freshwaters in the absence of new invading specialist degraders, but this idea remains untested. As generalist strategies often involve metabolic costs, such as a lower efficiency in exploiting specific resources in exchange for broader resource use, an increased dominance of generalists may reduce ecosystem productivity and trophic energy transfer [18]. The resulting shifts in bacterial composition from specialists to generalists may also increase the persistence of some compounds in the environment, creating feedbacks between climate-driven shifts in DOM composition and bacterial community composition. Although past studies rely on statistical correlations to infer the connections between DOM composition and different microbial communities [15, 19–21], they have not tracked how these connections change when the same microbes are exposed to natural DOM that varies in its bioavailability. Understanding how individual bacteria respond to different DOM sources is necessary to predict how climate change will change the persistence and transfer of organic carbon throughout freshwaters.

In this study, we tested how aquatic bacteria respond to diverse DOM substrates that span a gradient of bioavailability representative of future climate change and characterized feedbacks to the molecular composition of DOM from bacterial activity. While previous experiments have examined how complex bacterial communities assemble in simple resource environments, consisting of only a small number of organic compounds [22–24], here we grew a simplified, six-species bacterial community on natural DOM to tease apart the effect of resource complexity on individual species responses. Based on resource use assays, we classified five of the species as resource generalists and the sixth as a resource specialist, consistent with the observation that generalist species tend to dominate aquatic microbial communities [25]. We grew these communities on seven DOM sources. The sources ranged from fresh labile DOM, which resembled the ecological niche of five of the six bacterial species [26], to recalcitrant DOM, which was dominated by high molecular weight and aromatic compounds with lower bioavailability expected under future climate change [11]. We added the DOM to bacterial growth media as the sole carbon source at an ecologically relevant concentration, differing from past community assembly experiments that expose bacteria to simple carbon substrates at higher concentrations than observed in nature [24, 27]. We tracked changes in microbial community and DOM composition, as well as bacterial densities, at four intervals over 14 days using full-length amplicon 16S sequencing, ultra-high-resolution mass spectrometry, and flow cytometry, respectively. We hypothesized that shifts in microbial community composition arising from specialization for different DOM substrates feed back to change the bioavailability and persistence of aquatic carbon. By combining molecular and chemical datasets, our study now enables us to link bacterial community turnover with DOM processing to improve our understanding of the future functioning of freshwater environments.

## Materials and methods

### Dissolved organic matter preparation

We obtained DOM from seven contrasting sources. Six were collected from freshwater environments: a subarctic river (Northwest Territories, Canada; 62° 29′ 33.8″ N 113° 32′ 53.5″ W), subarctic lake (Northwest Territories, Canada; 62° 43′ 37.6″ N 115° 39′ 17.0″ W), boreal lake (Ontario, Canada; 46° 22′ 16.7″ N 81° 03′ 07.6″ W), temperate marsh (Ontario, Canada; 43° 54′ 17.2″ N 80° 24′ 34.5″ W), temperate pond (Cambridgeshire, UK; 52° 12′ 28.9″ N 0° 06′ 04.9″ E), and subtropical river (Georgia, USA; 30° 50′ 27.4″ N 82° 25′ 48.2″ W). The seventh source was prepared from beech leaf leachate, the standard carbon source used to culture five of the six experimental species [26, 28]. Before creating the experimental growth media, we concentrated DOM from each source using solid-phase extraction (SPE) [29]. Although SPE selectively excludes low molecular weight polar compounds [30], it ensured that the entire fraction of DOM that the bacteria grew upon would fall within the analytical window of the mass spectrometer. See Supplementary Methods for full DOM preparation details.

### Bacterial community preparation

We assembled a six-species bacterial community on each DOM source. Five species (*Arthrobacter* sp., *Pantoea* sp., *Pseudomonas fragi*, *Raoultella* sp., *Sphingobacterium* sp.) were isolated from a naturally co-occurring aquatic bacterial community that grows in accumulated rainwater at the base of beech trees (*Fagus sylvatica*) [28, 31]. These communities have been used extensively to test how microbial interactions shape community responses to novel environments [32, 33] and shift resource use during community succession [34] and evolution [28, 35]. As these bacteria are typically cultured on a complex carbon substrate (beech leaf leachate), they provide an ideal study system to test microbial community responses to diverse DOM. The sixth species, *Pseudomonas fluorescens* SBW25, was included because it is a model organism for microbial adaptation studies that are typically undertaken in liquid media [36]. These six species were selected because they represent different taxonomic families and they displayed different niche breadths based on carbon use assays (Fig. S1). Using these latter data, we classified *Sphingobacterium* as a specialist because it degraded the fewest carbon substrates (~20%), whereas the other species were classified as generalists because they can utilize a range of substrates across compound types (Fig. S1).

To create the experimental inoculum, we first thawed the bacteria from a frozen (−80°C) culture archive under sterile conditions and grew each species in monoculture on modified minimal M9 media (25% salts, glycerol at 3 mg C l^−1^) for 2 days at 22°C in the dark to allow for acclimation to laboratory conditions. The community was then assembled by combining each species at equal optical density (0.5 OD at 595 nm), measured using a spectrophotometer (Jenway 7300; Cole-Parmer, USA). Optical density depends on cell size and shape and does not always relate directly to cell count [37]. Consequently, our six species were combined at approximately equal biomass, but not total number of cells.

### Incubation experimental design

We prepared the bacterial media by adding each SPE DOM concentrate to modified M9 minimal media (25% salts; see Supplementary Methods) as the sole carbon source at a concentration of 4 mg C l^−1^. This concentration is nearly identical to the global average estimated for lakes with a surface area >0.1 km^2^ [38]. For each source, we prepared four pre-combusted (5 h, 500°C) 1-l bottles with 700 ml of media under sterile conditions. The initial pH across all bottles was a mean ± standard deviation (SD) of 7.39 ± 0.08. To start the experiment, we added 700 μl of the bacterial community inoculum to three replicate bottles for each DOM source and 700 μl of sterile MilliQ water to the fourth replicate as a negative control (total *n* = 28). The bottles were sealed gastight with sterile (autoclaved) bromobutyl rubber stoppers and incubated in the dark at 22°C.

We sampled the bottles in sterile conditions after 1, 4, 7, and 14 days. Each bottle was mixed, and we removed 100 ml of media with a sterile syringe and passed 95 ml through a 0.22-μm Sterivex filter (Millipore, USA) that was pre-rinsed with 180 ml of sterile MilliQ water. We collected the flow-through in pre-combusted amber glass vials, acidified the samples to pH 2 with HCl, and stored them at 4°C for DOM extraction and concentration measurements. The Sterivex filters were stored at −80°C for DNA extraction. The remaining 5 ml was fixed with glutaraldehyde (0.8% final concentration) in a cryotube, incubated at 4°C for 30 min, then flash frozen in liquid nitrogen and preserved at −80°C for cell counts.

### DNA sequencing

We used 16S rRNA gene metabarcoding to characterize how bacterial community composition varied with DOM source and time. DNA was first extracted from the filter cartridges following an established protocol modified from [39] (Supplementary Methods). We then amplified the entire 16S rRNA gene in each sample with the Oxford Nanopore Technologies (ONT) rapid barcoding kit SQK-RAB204 (ONT, UK) and followed the manufacturer’s protocol to prepare amplicons with the 27F–1492R primer pair (see Supplementary Methods). Polymerase chain reaction (PCR) products were pooled into libraries following the SQK-16S024 Flongle protocol (ONT, UK). A separate Flongle flow cell was loaded for each library and run for 24 h. We obtained between 4313 and 36 034 reads per sample (mean ± SD: 16 589 ± 9374). Negative controls (i.e. reagents only) and a positive control (i.e. ZymoBIOMICS® Microbial Community Standard II Log Distribution; Zymo Research, USA) were processed alongside the samples and confirmed that there was no contamination (see Supplementary Methods).

### Bioinformatics

Raw sequencing reads were base-called using *Guppy* version 6.0.6 (ONT, UK), discarding reads with *Q* ≤7, which is conservative for ONT data [40, 41]. We used *Guppy Barcoder* to remove barcode and adapter sequences from each read. We retained reads between 1300 and 1600 bases using *Nanofilt* [42] v.2.8.0, which corresponded to 86%–98% of reads per sample. Reads were aligned to a custom 16S rRNA reference database for our six species using *minimap2* [43] with default settings. The database was created from published whole-genome assemblies (Supplementary Methods). We retained only the primary alignments using *samtools* v.1.9 [44] and used the ‘merfePAF.py’ script of the Spaghetti pipeline [45] to summarize read mapping. Overall, we retained 3917–33 463 reads per sample (mean ± SD: 15 473 ± 8686). Raw reads from the microbial community standard were processed similarly, but mapped to the NCBI 16S + 18S rRNA database using the EPI2ME ‘Fastq 16S’ workflow (ONT, UK). We sequenced the microbial standard for each set of prepared libraries (*n* = 4) and pooled reads before alignment with EPI2ME. The composition of the positive control matched the expected theoretical distribution (Fig. S2), suggesting that our DNA extraction, sequencing, and bioinformatic methods generated expected and accurate results.

### Cell-density measurements

We determined cell concentrations in each sample using flow cytometry [46] with SYBR Green I (Thermo Fisher Scientific, USA) staining (see Supplementary Methods). No bacteria were detected in any control (i.e. no bacteria) bottles, suggesting no external contamination.

### Organic matter characterization

We measured the DOM concentration of every sample on a TOC-L analyser (Shimadzu, Japan). To characterize the molecular composition of DOM, we used a Vanquish Horizon ultra-high-performance liquid chromatograph coupled with a Q Exactive Orbitrap mass spectrometer (Thermo Fisher Scientific, USA) run in negative ionization mode (instrument settings are given in Supplementary Methods) [47]. Every sample underwent SPE before analysis (see Supplementary Methods), with a mean ± SD extraction efficiency of 58.4 ± 7.0% (*n* = 56).

We assigned formulae to mass lists using functions from *MFAssignR* [48] in R v.4.2.1 [49] (see Supplementary Methods). We retained formulae identified in at least two of three replicates for each sampling day and DOM source combination. The final dataset contained 6478 formulae. For each sample, we normalized peak intensities by the total intensity of the sample and calculated average intensity-weighted bulk DOM metrics [50]: degree of saturation (the ratio of hydrogen to carbon atoms, H/C, with larger values indicating greater bioavailability) [51, 52], oxidation state (the oxygen to carbon ratio, O/C) [51], and modified aromaticity index (larger values indicating greater aromaticity, AI_mod_) [53]. We also summed the relative intensity of bioavailable formulae, defined as those with an H/C ratio ≥1.5 [52]. Finally, we used established criteria to group formulae into putative compound classes based on their H/C and O/C ratios [54] (see Supplementary Methods).

### Statistical analyses

We first tested if the composition of bacterial communities and DOM sources varied after 14 days using permutational multivariate analysis of variance (PERMANOVA) estimated using the ‘adonis2’ function in the R package *vegan* [55] with default settings (Bray–Curtis dissimilarities, 999 permutations). To identify variables that contributed to the observed variation, we used the ‘envfit’ function in *vegan* to correlate the first two axes of separate principal coordinate analyses (PCoA) of Bray–Curtis dissimilarities for bacteria and DOM composition with bulk DOM metrics, the summed relative intensity of each compound class, and the relative abundance of each bacterial species. We tested if bacterial community composition covaried with DOM composition by correlating the two PCoAs using the ‘procrustes’ function in *vegan* and tested statistical significance using 999 randomized permutations. We further tested the effect of DOM composition on bacterial community composition using a distance-based redundancy analysis (dbRDA) implemented with the ‘dbrda’ function in *vegan* (Supplementary Methods).

We then asked if the composition of bacteria communities and DOM changed over time. We analysed communities as a whole using PERMANOVA (see Supplementary Methods). We then tested how the relative abundance of each bacterial species, the intensity-weighted relative abundances of each compound class, and the bulk DOM metrics changed over time within each source using generalized linear models. All models included time as a predictor with quasibinomial errors to account for overdispersion in bacterial counts. To assess temporal trends in cell densities (log-transformed) and DOM concentrations, we fitted linear mixed-effects models with time and DOM source as predictors, and accounted for random variation due to repeated measurement of the same replicate.

We also examined changes in individual formulae. We used Spearman’s rank correlations to determine if the relative abundance of each molecular formula varied with time. Only formulae present in ≥2 replicates at every time point were analysed and the temperate pond was excluded due to missing data. We focused on the strongest correlations in the dataset to identify formulae that had the greatest changes during the experiment. We defined the strongest positive and negative correlations as those with an absolute correlation coefficient in the top 5% of all correlations, corresponding to |ρ| ≥0.750. The null expectation was that formulae within this top 5% were split equally between positive and negative correlations within each compound class [56]. More correlations than expected under the null hypothesis indicates that the compound class was preferentially consumed (negative correlations) or produced (positive correlations). We used an exact multinomial test implemented with the *EMT* package [57] to test this null hypothesis. For compound classes with a statistically significant deviation from expected (*P* < .05), we used a *post hoc* exact binomial test to determine the direction of the effects.

To characterize putative bacterial resource use, we correlated the relative abundance of each species with the relative abundance of every molecular formula across the time series on each DOM source using Spearman’s rank correlations. We focused on the strongest 2.5% of negative correlations across the DOM sources, corresponding to a ρ ≤−0.706, which we hypothesized were indicative of resource use [58]. We then asked if the species differed in overall resource use. We tested if the probability of a formula being utilized varied among species by fitting a mixed-effects model with a binomial error structure to the observed number of negative and positive correlations estimated between every compound class and species on each DOM source. Species was the sole predictor in the model, and we accounted for random variation due to repeated measurement of the same compound class and DOM source. Lastly, we asked if formulae that correlated negatively with each bacterial species decreased over time. We identified formulae within the top 2.5% negative correlations with time, corresponding to ρ ≤−0.806. For each DOM source and species, we summed the total number of formulae whose relative abundance both correlated negatively with the bacterial abundance and decreased over time to characterize putative resource use of species.

## Results

### DOM sources represented a bioavailability gradient

**Figure 1:**
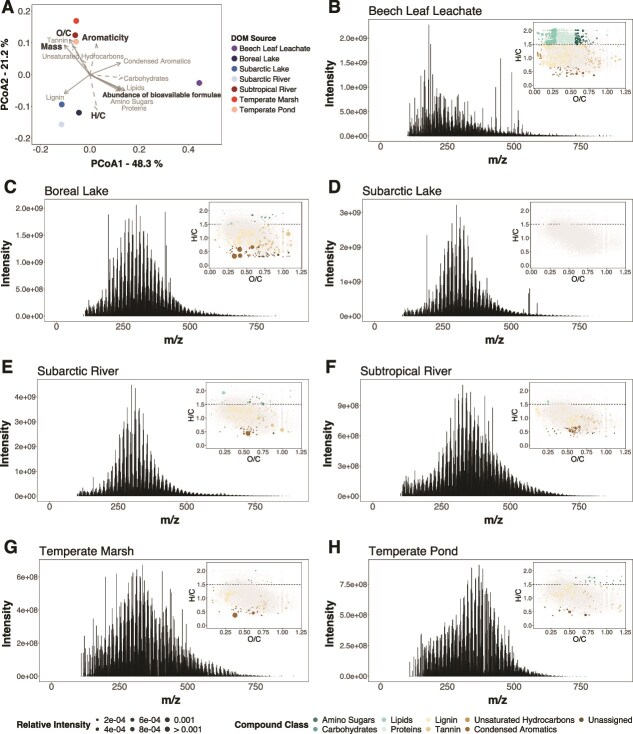
DOM sources represented a gradient of bioavailability and clustered into three distinct groups based on molecular composition. (**A**) Principal coordinate analysis (PCoA) of the Bray–Curtis dissimilarities of formulae assigned to each of the seven DOM sources. DOM composition from the initial media of each source was characterized using mass spectrometry. Due to missing data for the initial temperate marsh media, the day 1 no-bacteria control sample from the experiment was used to characterize this source. Vectors summarize the molecular composition of the samples, with solid lines indicating statistically significantly correlations with the ordination (*P* < .05). Vectors included average intensity-weighted H/C, O/C, aromaticity (modified aromaticity index), mass (mass-to-charge ratio), the abundance of bioavailable formulae (relative intensity of formulae with H/C ratio > 1.5), and the relative abundance of formulae from each putative compound class. Samples sitting near the heads of the arrows had greater values for the corresponding molecular properties and higher relative abundances of each compound class. Percentages indicate variation explained by each PCoA axis. The DOM sources cluster into three groups based on composition: fresh labile DOM (beech leaf leachate), worked labile DOM (boreal lake, subarctic pond, subarctic river), and recalcitrant DOM (subtropical river, temperate marsh, temperate pond). (**B**)–(**H**) Spectra showing the mass-to-charge ratio (*m*/*z*) and intensity of molecular formula identified within each DOM source. Inlay panels show a van Krevelen diagram for each DOM source, with each formula plotted based on the H/C and O/C ratios and circle sizes indicating relative intensity. Formulae that are unique to the given DOM source are coloured according to the putative compound class. The dashed line at H/C = 1.5 indicates the biolability boundary [52]. Total number of molecular formulae assigned to each source: beech leaf leachate—3969; boreal lake—3374; subarctic lake—2916; subarctic river—3757; subtropical river—3246; temperate marsh—2792; temperate pond—2649.

The seven DOM sources were compositionally distinct and clustered into three distinct groups based on chemical composition (Fig. 1A): fresh labile, worked labile, and recalcitrant DOM. The beech leaf leachate (fresh labile DOM) contained the highest relative abundance of bioavailable formulae (i.e. H/C > 1.5) and the lowest average molecular weight (Fig. 1B). This source also contained relatively high abundances of labile compounds, e.g. amino sugars, carbohydrates, lipids, and proteins. The boreal lake, subarctic lake, and subarctic river (worked labile DOM) were characterized by high average H/C ratios, but lower relative abundances of labile compounds compared to the beech leachate. These sources contained the highest relative abundances of lignin-like compounds (Fig. 1C–E). In contrast, the subtropical river, temperate marsh, and temperate pond (recalcitrant DOM) contained the highest relative abundances of oxidized, high molecular weight, and aromatic compounds, with low abundance of labile formulae (Fig. 1F–H).

### DOM composition structured bacterial communities

**Figure 2:**
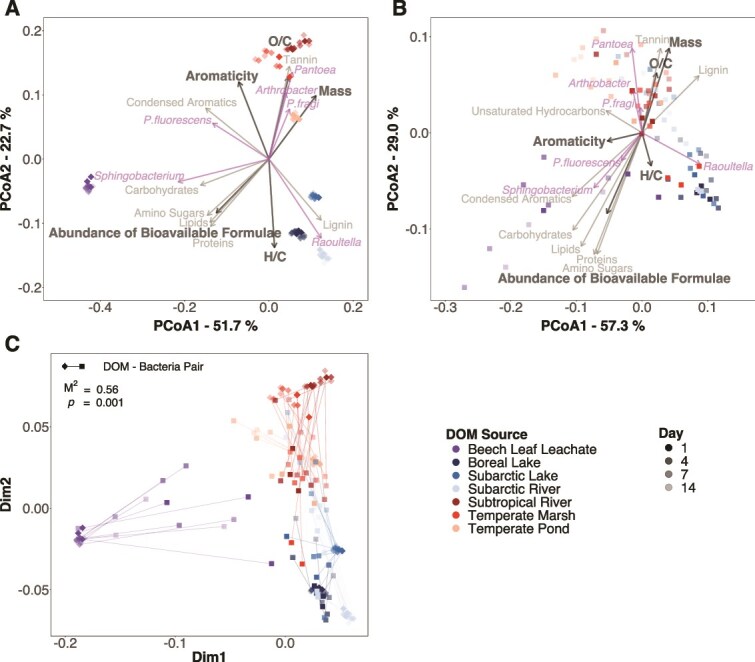
Communities of molecular formulae and bacteria covaried across contrasting DOM sources. We fitted a separate principal coordinate analysis (PCoA) to the relative abundances of (**A**) molecular formulae and (**B**) bacterial species measured in triplicate on seven DOM sources at four time points (total *n* = 80 and 84 in (**A**) and (**B**), respectively; values vary due to missing molecular data on the temperate pond). Each point is a sample, with the distance between points related to sample similarity. Points that are closer together are more similar in their relative abundances of (**A**) molecular formulae and (**B**) bacterial species. For each sample, we characterized bulk molecular composition by calculating the average intensity-weighted H/C ratio, O/C ratio, aromaticity (modified aromaticity index), mass (mass-to-charge ratio), and abundance of bioavailable formulae (relative intensity of formulae with H/C ratio > 1.5). Vectors summarize the molecular and species composition of samples across the dataset, with samples sitting near the heads of the arrows having greater values, on average, for the corresponding DOM metric and higher relative abundances of each species/compound class. All vectors had statistically significantly correlations with the ordinations (*P* < .05). Percentages indicate variation explained by each PCoA axis. (**C**) To test the strength of the association between the DOM and bacterial community compositions, we used a Procrustes analysis, which overlays the PCoA ordinations in (**A**) and (**B**) and rotates the axes to maximize congruence between them. Each sample is therefore represented by a line connecting the DOM composition (diamond) with bacterial community composition (triangle) (total *n* = 80).

**Figure 3:**
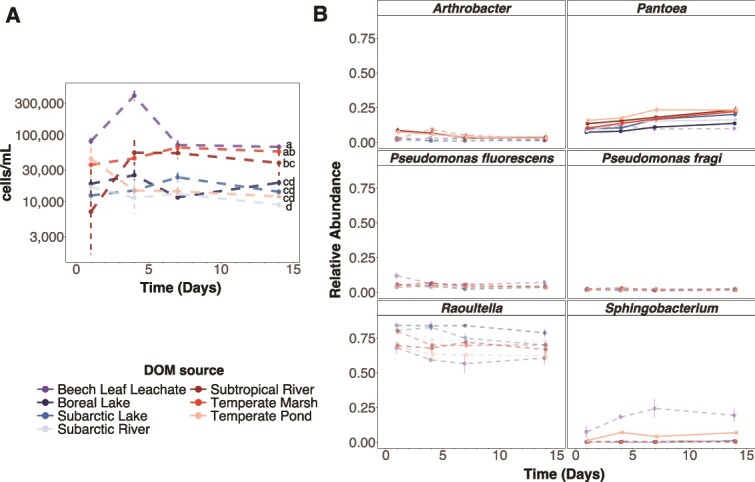
Total cell densities remained constant while *Pantoea* consistently increased in relative abundance over time. Points are (**A**) the mean cell density (cells/ml) ± standard error of the bacterial community or (**B**) the mean relative abundance ± standard error of each species based on 16S rRNA sequence data across three replicate communities on each of the seven DOM sources. Different letters in (**A**) indicate statistically significant differences in the average cell density between DOM sources based on a *post hoc* Tukey test (*P* < .05). There was no interaction between DOM source and time (ANOVA: *F*_6,56_ = 1.96, *P* = .086), so the interaction was dropped from the model before running the *post hoc* Tukey test. Solid lines in (**B**) indicate statistically significantly changes in the relative abundance of the species over time (Table S3).

We found evidence that DOM composition structured bacterial communities across the 14-day incubation. Bacterial community composition diverged among DOM sources across all time points (PERMANOVA: *F* = 19.1, *P* = .001; Fig. 2; Table S1) with DOM composition explaining 67.3% of the variation in microbial community composition (dbRDA: *F* = 10.44, *P* = .001; Fig. S3). After 1 day, the mean ± SD for the Bray–Curtis community dissimilarity across DOM sources was 11.5 ± 6.1%, which was nearly twice the dissimilarity among replicates within each DOM source (6.2 ± 4.5%; *t*_29.02_ = 4.90, *P* < .001). This difference persisted until the end of the experiment, with 13.4 ± 7.7% dissimilarity among DOM sources at day 14 compared to 7.1 ± 3.7% within each source (*t*_43.4_ = 6.52, *P* < .001). The DOM sources also supported different cell densities (cells ml^−1^) according to flow cytometry (ANOVA: *F*_6,59.4_ = 6.08, *P* < .001; Fig. 3A). Bacteria had 1.7 to 9.0 times higher cell densities, on average, on the beech leachate, temperate marsh, and subtropical river than the other sources (Fig. 3A).

*Raoultella* dominated all communities, ranging from a mean ± SD of 61.1 ± 8.1% of reads on the beech leachate to 82.6 ± 3.8% of reads on the boreal lake (Fig. S4). However, all six species were always detected (Fig. S4), suggesting some coexistence. Different species grouped with different DOM clusters in the PCoA (Fig. 2A–B; Table S2), likely because of resource preferences. *Sphingobacterium* reached the highest relative abundance on the labile beech leachate, with a mean of 17.3 ± 9.4% of reads (Fig. S4), and was associated with a higher abundance of bioavailable compounds (i.e. clustered near arrowheads of bioavailable formulae in Fig. 2A). *Raoultella* was the most abundant on the lignin-dominated worked labile DOM (boreal lake, subarctic, subarctic river; Fig. 2A–B), while *Pantoea*, *Arthrobacter*, and *P. fragi* were associated with the recalcitrant DOM sources that contained greater proportions of tannin-like formulae (temperate pond, temperate marsh, subtropical river; Fig. 2A–B). Overall, molecular and bacterial community compositions were strongly correlated across all DOM sources (Procrustes: *M*^2^ = 0.56, *P* = .001; Fig. 2C), indicating that substrates with similar chemical compositions supported similar bacterial communities.

### Bacterial community composition changed similarly over time across sources

Temporal changes in bacterial community composition were largely consistent across substrates. Community composition changed across the 14 days on all sources except beech leachate and changed similarly over time based on PERMANOVAs (Table S1). We attributed the temporal changes to consistent increases among DOM sources in the generalist *Pantoea*, which nearly doubled in relative abundance from a mean ± SD of 10.8 ± 3.3% on day 1 to 19.8 ± 4.4% by day 14 on all sources except the beech leachate (Fig. 3B, Table S3). Only two other species changed in abundance (Fig. 3B; Table S3). *Sphingobacterium* increased from 0.5 ± 0.6% on day 1 to 2.7 ± 3.1% relative abundance by day 14 on two worked labile sources (boreal lake, subarctic river) and one recalcitrant source (temperate pond) (Fig. 3B, Table S3). In contrast, *Arthrobacter* decreased in abundance from 8.1 ± 3.0% on day 1 to 3.4 ± 1.7% by day 14 on two recalcitrant sources (temperate pond, subtropical river) (Fig. 3B, Table S3). Finally, we observed that community cell densities were largely constant over time within each source (ANOVA: *F*_1,56_ = 0.23, *P* = .631), suggesting that communities quickly reached equilibrium specific to each DOM source and were turning over in composition (Fig. 3A).

### Reworking of DOM promoted less bioavailable formulae

We observed small changes in bulk composition of DOM. Three labile sources (beech leachate, subarctic river, subarctic lake) and two recalcitrant source (temperate marsh, temperate pond) changed in composition over 14 days in different ways according to PERMANOVAs (Table S4). However, we detected few and mostly weak (<10% in magnitude) changes in the relative abundances of compound classes and bulk DOM metrics (Tables S5 and S6; Fig. S5). Additionally, DOM concentrations remained similar over time across sources (ANOVA: *F*_1,54.2_ = 0.07, *P* = .790; Fig. S6), with a mean ± SD decrease of 0.25 ± 3.9% after 14 days, suggesting that microbial reworking of DOM removed little carbon during this experiment.

**Figure 4:**
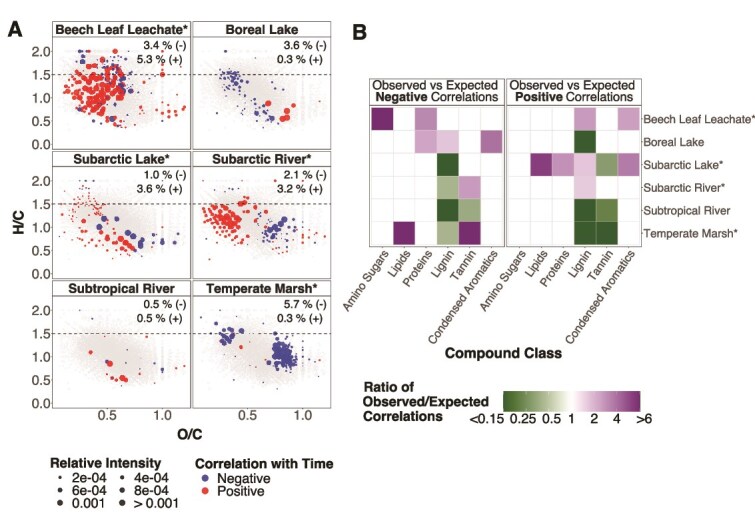
Relative intensity of less bioavailable compounds increased following reworking of labile substrates. We calculated a Spearman’s rank correlation between the relative intensity of each molecular formula and time to identify formulae that increased or decreased over the 14-day incubation, which we hypothesize represent compounds that were produced and consumed, respectively. We focused on correlations in the top 5% across the dataset (|ρ| ≥ 0.750). (**A**) Molecular formulae (i.e. each point) on each DOM source are sized based on their relative intensity at day 1 averaged across three replicates. Coloured points show formulae that were positively and negatively correlated with time and are sized based on their relative intensity at day 14 averaged across the three replicates. The dashed line at H/C = 1.5 indicates the biolability boundary [52]. DOM sources are arranged based on high (beech leaf leachate, boreal lake, subarctic river, subarctic lake) and low lability (subtropical river, temperate marsh). The temperate pond was excluded due to missing molecular data. Numbers in the top-right corner indicate the percentage of formulae that were positively (+) and negatively (−) correlated with time on each DOM source. (**B**) After assigning each formula to putative compound classes, we used a multinomial test to determine if the number of compounds that increased or decreased over time within each compound class differed from expected. A higher number of negative and positive correlations than expected under the null hypothesis indicates that the compound class is preferentially consumed and produced by the bacteria, respectively. Coloured squares indicate compound classes with increasingly more or less formulae correlated with time than expected. We excluded carbohydrates and unsaturated hydrocarbons because these did not differ from null expectations on any DOM sources. * indicates sources that changed in overall composition over time based on a PERMANOVA (Table S4).

Despite small changes in bulk DOM, microbial reworking clearly influenced individual molecular formulae. On the three labile DOM sources that changed in bulk composition, temporal changes in DOM composition were mostly due to less bioavailable formulae (i.e. H/C < 1.5) increasing in relative abundance (Fig. 4A). Overall, 71.7%, 82.6%, and 58.8% of the less bioavailable formulae that changed with time increased in abundance on the beech leachate, subarctic lake, and subarctic river, respectively. Of these, more lignin- and condensed aromatic-like formulae were positively correlated with time than expected by chance (exact binomial test: *P* < .004; purple in Fig. 4B). These less bioavailable compounds may have increased in relative abundance as bioavailable compounds were preferentially consumed by the microbes, as indicated by more amino sugar- and protein-like compounds negatively correlating with time on the beech leachate than expected (*P* < .001; Fig. 4B). Additionally, on the subarctic lake and subarctic river, we found fewer lignin-like compounds than expected correlated negatively with time (Fig. 4B), suggesting that bacteria did not readily consume these recalcitrant substrates.

In addition to consuming bioavailable substrates, microbial reworking also produced bioavailable compounds on one labile source. On the subarctic lake, we observed nearly two to three times more positive correlations with time than expected for protein- and lipid-like compounds (*P* < .003, Fig. 4B), suggesting that bacteria were producing these compounds, such as through exudation. However, this increase was not large enough to offset the increase in abundance of less bioavailable compounds. Protein- and lipid-like compounds accounted for only 0.3% of the total intensity, while the less bioavailable lignin- and condensed aromatic-like formulae that increased in relative abundance accounted for 2.5% of total intensity on this source.

In contrast to the labile DOM, microbial reworking on the temperate marsh led to a decrease in the relative abundance of less bioavailable formula (Fig. 4A). Of the less bioavailable formulae that changed with time, 94.7% decreased in relative abundance, with >6 times more tannin-like compounds decreasing than expected (Fig. 4B). The bacterial community also appeared to preferentially consume lipid-like formulae on this DOM source, while no compound classes were positively correlated with time more than expected by chance (Fig. 4B).

### The generalist *Pantoea* had the greatest putative resource use

**Figure 5:**
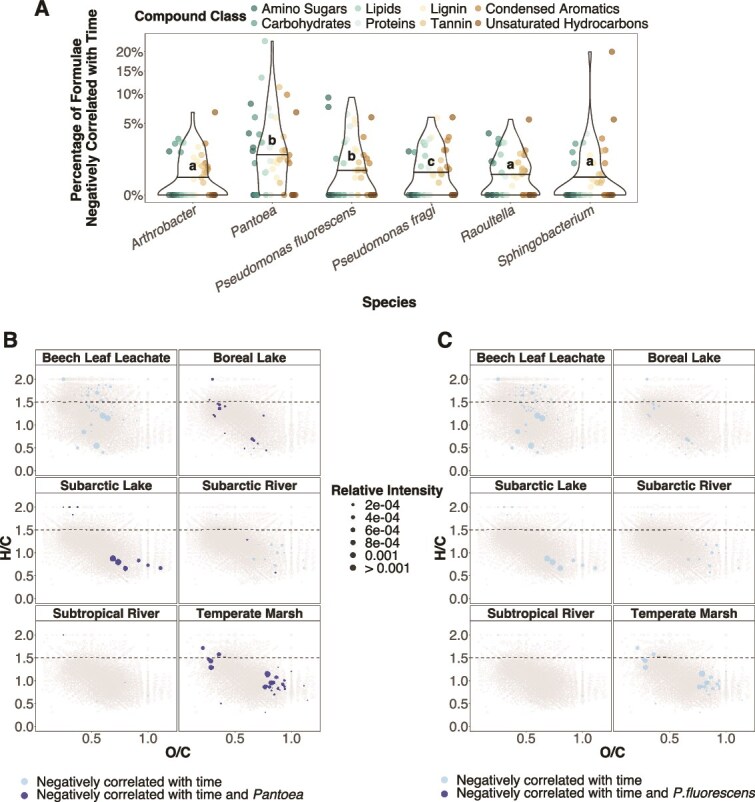
*Pantoea* had the greatest putative resource use among the six bacterial species. (**A**) Relative abundance of each bacterial species within the community was correlated with the relative intensity of every molecular formula across 14 days. The most negative 2.5% of correlations, corresponding to a Spearman ρ ≤ −0.806, were considered to reflect resource use. For each bacterial species, we then calculated the proportion of utilized formula within each compound class on every DOM source (*n* = 288). The violin plots show the distribution of these data, with the horizontal lines showing the mean percentage of formulae consumed across compound classes for each species. The proportion of negative correlations varied by species according to a generalized mixed-effects model (*χ*^2^ = 248.12, *df* = 5, *P* < .001), with different letters indicating differences between species (*P* < .05) based on a Tukey *post hoc* test. The temperate pond source was excluded due to missing molecular data. We then identified molecular formulae that both correlated negatively with each species and correlated negatively with time across the incubation experiment. The van Krevelen diagrams in (**B**) and (**C**) show the results for *Pantoea* and *P. fluorescens*, respectively. Individual molecular formulae are represented by circles sized based on their relative intensity at day 1 averaged across three replicates per DOM source. Coloured points show formulae that were either strongly negatively correlated with time but not the focal species, or negatively correlated with both time and the focal species, sized based on the average relative intensity at day 14 across replicates. Negative correlations were defined as the strongest 2.5% of correlations, corresponding to ρ ≤ −0.706 in the dataset of species-by-formulae correlations and ρ ≤ −0.806 in the dataset of formulae-by-time correlations. The dashed line at H/C = 1.5 indicates the biolability boundary.

We found evidence that temporal changes in abundances of individual taxa reflected preferences for specific compounds. Based on how relative abundances of bacteria and molecular formulae correlated, *Pantoea* had the largest putative resource use, correlating negatively, on average, with the most formulae per compound class (mean ± SD: 2.9 ± 4.4%; Fig. 5A). Correlations with lipid- and tannin-like compounds were particularly high, with *Pantoea* correlating negatively with up to 23% and 11%, respectively, of formulae within these classes (Fig. 5A). Of all formulae that correlated negatively with *Pantoea*, 18.0% also decreased in relative abundance over time within the DOM sources on which *Pantoea* became more abundant (Fig. 5B). In contrast, *P. fluorescens* did not differ from *Pantoea* in mean putative resource use (1.4 ± 2.3%, Fig. 5A), but we found no evidence that it reduced the abundances of the compounds it utilized (Fig. 5C). *Arthrobacter* showed evidence of having the narrowest niche, i.e. correlated with the fewest molecular formulae (0.8 ± 1.3%; Fig. 5A), suggesting that it had the lowest relative abundances because it was competitively excluded on certain sources (Fig. 5B). There was little evidence that *Arthrobacter*, *P. fragi*, *Raoultella*, or *Sphingobacterium* reduced the abundances of compounds they utilized (Fig. S7).

## Discussion

Given the importance of bacteria for the functioning of freshwaters, understanding how they respond to shifts in organic substrate composition is essential to inform ecosystem management and modelling. Here, we showed that bacterial communities initially diverged in composition when grown on compositionally distinct DOM, but then changed similarly through time, reflecting their specialization for different resources. These results suggest that generalist bacteria with the broadest niches may emerge as ‘winners’ as climate change shifts DOM composition [59]. There were also feedbacks from bacteria to DOM that resulted in a negligible depletion of DOM concentration but a shift in its composition towards less bioavailable compounds. Overall, our study indicates that as bacteria encounter resources outside of their ecological niches, the loss of specialization, at least in the short term, may increase the persistence of DOM, with potential consequences for the trophic transfer of carbon through freshwaters. For example, shifts towards generalist bacteria may alter the nutritional quality of biomass available to zooplankton because bacteria differ markedly in biochemical composition and in how efficiently they are assimilated by grazers [60]. Total trophic transfer will also depend on bacterial densities, which we found was highest on the most recalcitrant DOM. Future work will need to disentangle the broader ecological consequences of changes in bacterial composition and abundance arising from shifting DOM [11].

Species sorting likely explains how DOM composition initially structured the microbial communities. We found more similar DOM sources supported more similar microbial communities, and we attributed these differences to the ability of species to utilize different carbon substrates. For example, *Arthrobacter*, *Pantoea*, and *P. fragi* generally reached higher abundances on recalcitrant DOM sources, as they are known to utilize higher molecular weight and oxidized compounds [61–63]. Similarly, the resource specialist *Sphingobacterium* may have only been competitive and reached its highest relative abundance on the DOM source that provided a niche matching its natural environment, i.e. beech leaf leachate [26, 28]. Although previous studies have also demonstrated that the composition of organic substrates structures bacterial communities [12, 17, 64], we now show that this structuring is consistent on different DOM sources with varying bioavailability.

Despite clear structuring of the bacterial communities by DOM composition, *Raoultella* dominated all communities. One explanation for this result is that the species were assembled in the community to achieve comparable biomass, not cell counts. Among the six species, *Raoultella* has the smallest estimated cell size (Table S7), so more cells were likely required to give the same biomass as the other strains, resulting in higher relative abundance within the starting community. An alternative explanation is that high relative abundances of *Raoultella* reflect a greater 16S rRNA gene copy number (Table S7). We did not correct for 16S copy number in our analysis, as it was unmeasured and doing so using estimated copy numbers from closely related species can introduce uncertainty [65]. Both *P. fragi* and *Sphingobacterium* have comparable copy numbers (Table S7), yet they never reached the same abundances as *Raoultella*, suggesting that copy number variation is an unlikely explanation for our results. Finally, *Raoultella* could have been more ecologically dominant, at least initially. We found that *Raoultella* used the greatest diversity of carbon substrates when grown in monoculture, consistent with observations where *Raoultella* dominated species mixtures grown on different sugars [24]. *Raoultella* may have instead been unable to increase in abundance over time as it did not have the largest putative resource use on our specific DOM sources.

Once established on the different DOM sources, the niche breadth of taxa predicted if they repeatedly increased in abundance over time. The broad niche of *Pantoea* likely gave it a competitive advantage over the other generalist species as bioavailable compounds were preferentially depleted [17]. This advantage may have declined on the beech leachate that most closely resembles the ecological niche of the community, explaining why the specialist *Sphingobacterium* grew. An alternative explanation for our results is that *Pantoea* increased in abundance because of a faster generation time. However, the decline in *Arthrobacter* coinciding with increases in *Pantoea* suggests at least some competitive displacement. More generally, these results indicate that temporal dynamics of bacterial communities cannot be entirely predicted from metabolic preferences of constituent members when they are grown in isolation. For example, *Pantoea* used fewer resources in monoculture than *Raoultella* based on assays of individual carbon substrates, but was the only species to increase consistently in abundance across the incubation experiment. Species interactions, such as competition, may have altered the realized niche breadths of taxa when grown in the community versus expectations from monoculture [16]. We note that by using a DNA-based approach to characterize relative abundances, we may have also sequenced some dead bacteria. Nonetheless, the potential inclusion of dead cells does not change our conclusions. The relative abundance of non-active cells would either remain constant over time or decline as cells lyse, enabling growth-related increases in the relative abundance of *Pantoea* to be detected. Overall, temporal dynamics of the communities were highly influenced by the resource breadth of a single species (i.e. *Pantoea*), irrespective of DOM composition.

Consistent with the observation that bacteria readily consume more bioavailable compounds [17, 52], we found that DOM sources converged towards a less bioavailable composition. Microbial reworking has been widely observed to result in less bioavailable or persistent DOM [66, 67]. However, the extent of metabolic diversity required to produce such a pattern has remained unclear. Here, we advance past studies [66, 67] by demonstrating that the same pattern as in nature emerged after just 14 days with only six species. The compositional shifts in DOM were not merely due to the preservation of persistent compounds because bioavailable protein-like and amino sugar–like compounds were depleted. Observing this process even with just six species suggests that it may generalize across higher-order communities with much greater metabolic diversity [68]. Therefore, as global change restructures microbial communities [69], DOM may predictably become reworked independent of metabolic or taxonomic diversity.

Despite evidence of microbial reworking, bulk DOM concentration did not decrease over time like in other incubation experiments [70–73]. One explanation is that insufficient time elapsed to detect a difference. A synthesis of incubation experiments examining the degradation of aquatic DOM found that across ecosystems, the labile fraction only accounts for an average 6.1% of the total DOM pool and is readily consumed within 31 days [74]. A 6.1% decrease in DOM concentration matches the declines we observed over 14 days on our most labile source and is greater than observed on our other sources. A second possibility is that SPE removed many small bioavailable compounds, such as sugars, which bacteria readily consume [30, 75].

Our study suggests that bacterial communities can shift towards generalist taxa as they face novel ecological conditions, potentially increasing the persistence of aquatic carbon. Although our bacterial community was less diverse than natural aquatic microbiomes, reducing the community to only six species allowed us to characterize the resource use and temporal responses of individual strains to diverse DOM substrates, as well as the implications for DOM persistence. When applied to more complex communities, our approach to combine compositional time series from bacteria and DOM may ultimately improve estimates of resource use and better predict microbial temporal dynamics in aquatic environments.

## Supplementary Material

Sandor_ISMECOMMUN-D-25-00556R2_supplement-final_ycag045

## Data Availability

The 16S rRNA gene sequences were deposited into the European Nucleotide Archive (ENA) under project accession PRJEB105935. Data files and analysis scripts are available in the Figshare repository at the following link: https://figshare.com/s/e281b3501e23ddc4f739.

## References

[1] Berggren M, Ström L, Laudon H et al. Lake secondary production fueled by rapid transfer of low molecular weight organic carbon from terrestrial sources to aquatic consumers. *Ecol Lett* 2010;13:870–80. 10.1111/j.1461-0248.2010.01483.x 20482576

[2] Solomon CT, Jones SE, Weidel BC et al. Ecosystem consequences of changing inputs of terrestrial dissolved organic matter to lakes: current knowledge and future challenges. *Ecosystems* 2015;18:376–89. 10.1007/s10021-015-9848-y

[3] Berggren M, Guillemette F, Bieroza M et al. Unified understanding of intrinsic and extrinsic controls of dissolved organic carbon reactivity in aquatic ecosystems. *Ecology* 2022;103:e3763. 10.1002/ecy.3763 35612376 PMC9540823

[4] Tanentzap AJ, Fonvielle JA. Chemodiversity in freshwater health. *Science* 2024;383:1412–4. 10.1126/science.adg8658 38547265

[5] Hartnett HE . Dissolved organic matter (DOM). In: White W.M. (ed.), Encyclopedia of Geochemistry. Encyclopedia of Earth Sciences Series. Cham: Springer, 2018, 375–8.

[6] Guillemette F, McCallister SL, del Giorgio PA. Selective consumption and metabolic allocation of terrestrial and algal carbon determine allochthony in lake bacteria. *ISME J* 2016;10:1373–82. 10.1038/ismej.2015.215 26623544 PMC5029189

[7] Ortega-Retuerta E, Devresse Q, Caparros J et al. Dissolved organic matter released by two marine heterotrophic bacterial strains and its bioavailability for natural prokaryotic communities. *Environ Microbiol* 2021;23:1363–78. 10.1111/1462-2920.15306 33185969

[8] Taipale SJ, Rigaud C, Calderini ML et al. The second life of terrestrial and plastic carbon as nutritionally valuable food for aquatic consumers. *Ecol Lett* 2023;26:1336–47. 10.1111/ele.14244 37218115

[9] Tanentzap AJ, Szkokan-Emilson EJ, Kielstra BW et al. Forests fuel fish growth in freshwater deltas. *Nat Commun* 2014;5:4077. 10.1038/ncomms5077 24915965 PMC4082636

[10] Jones SE, Solomon CT, Weidel BC. Subsidy or subtraction: how do terrestrial inputs influence consumer production in lakes? *Fr Rev* 2012;5:37–49. 10.1608/FRJ-5.1.475

[11] Creed IF, Bergström A-K, Trick CG et al. Global change-driven effects on dissolved organic matter composition: implications for food webs of northern lakes. *Glob Chang Biol* 2018;24:3692–714. 10.1111/gcb.14129 29543363

[12] Roiha T, Peura S, Cusson M et al. Allochthonous carbon is a major regulator to bacterial growth and community composition in subarctic freshwaters. *Sci Rep* 2016;6:34456. 10.1038/srep34456 27686416 PMC5043279

[13] Wang Y, Xie R, Shen Y et al. Linking microbial population succession and DOM molecular changes in *Synechococcus*-derived organic matter addition incubation. *Microbiol Spectrum* 2022;10:e02308–21. 10.1128/spectrum.02308-21 PMC9045170 35380472

[14] Gómez-Consarnau L, Lindh MV, Gasol JM et al. Structuring of bacterioplankton communities by specific dissolved organic carbon compounds. *Environ Microbiol* 2012;14:2361–78. 10.1111/j.1462-2920.2012.02804.x 22697392

[15] Ávila MP, Brandão LPM, Brighenti LS et al. Linking shifts in bacterial community with changes in dissolved organic matter pool in a tropical lake. *Sci Total Environ* 2019;672:990–1003. 10.1016/j.scitotenv.2019.04.033 30981171

[16] Bell TH, Bell T. Many roads to bacterial generalism. *FEMS Microbiol Ecol* 2021;97:fiaa240. 10.1093/femsec/fiaa240 33238305

[17] Wu X, Wu L, Liu Y et al. Microbial interactions with dissolved organic matter drive carbon dynamics and community succession. *Front Microbiol* 2018;9:1234. 10.3389/fmicb.2018.01234 29937762 PMC6002664

[18] Gravel D, Bell T, Barbera C et al. Experimental niche evolution alters the strength of the diversity-productivity relationship. *Nature* 2011;469:89–92. 10.1038/nature09592 21131946

[19] Chen Q, Chen F, Gonsior M et al. Correspondence between DOM molecules and microbial community in a subtropical coastal estuary on a spatiotemporal scale. *Environ Int* 2021;154:106558. 10.1016/j.envint.2021.106558 33878614

[20] Osterholz H, Kirchman DL, Niggemann J et al. Diversity of bacterial communities and dissolved organic matter in a temperate estuary. *FEMS Microbiol Ecol* 2018;94:fiy119. 10.1093/FEMSEC/FIY119 29912327

[21] Tanentzap AJ, Fitch A, Orland C et al. Chemical and microbial diversity covary in fresh water to influence ecosystem functioning. *Proc Natl Acad Sci USA* 2019;116:24689–95. 10.1073/pnas.1904896116 31740592 PMC6900631

[22] Estrela S, Vila JCC, Lu N et al. Functional attractors in microbial community assembly. *Cell Syst* 2022;13:29–42.e7. 10.1016/j.cels.2021.09.011 34653368 PMC8800145

[23] Dal Bello M, Lee H, Goyal A et al. Resource–diversity relationships in bacterial communities reflect the network structure of microbial metabolism. *Nat Ecol Evol* 2021;5:1424–34. 10.1038/s41559-021-01535-8 34413507

[24] Goldford JE, Lu N, Bajić D et al. Emergent simplicity in microbial community assembly. *Science* 2018;361:469–74. DOI:10.1126/science.aat1168 30072533 PMC6405290

[25] von Meijenfeldt FAB, Hogeweg P, Dutilh BE. A social niche breadth score reveals niche range strategies of generalists and specialists. *Nat Ecol Evol* 2023;7:768–81. 10.1038/s41559-023-02027-7 37012375 PMC10172124

[26] Rivett DW, Bell T. Abundance determines the functional role of bacterial phylotypes in complex communities. *Nat Microbiol* 2018;3:767–72. 10.1038/s41564-018-0180-0 29915204 PMC6065991

[27] Estrela S, Sanchez-Gorostiaga A, Vila JCC et al. Nutrient dominance governs the assembly of microbial communities in mixed nutrient environments. *eLife* 2021;10:e65948. 10.7554/ELIFE.65948 33877964 PMC8057819

[28] Scheuerl T, Hopkins M, Nowell RW et al. Bacterial adaptation is constrained in complex communities. *Nat Commun* 2020;11:754. 10.1038/s41467-020-14570-z 32029713 PMC7005322

[29] Dittmar T, Koch B, Hertkorn N et al. A simple and efficient method for the solid-phase extraction of dissolved organic matter (SPE-DOM) from seawater. *Limnol Oceanogr Methods* 2008;6:230–5. 10.4319/lom.2008.6.230

[30] Raeke J, Lechtenfeld OJ, Wagner M et al. Selectivity of solid phase extraction of freshwater dissolved organic matter and its effect on ultrahigh resolution mass spectra. *Environ Sci Process Impacts* 2016;18:918–27. 10.1039/c6em00200e 27363664

[31] Bell T, Newman JA, Silverman BW et al. The contribution of species richness and composition to bacterial services. *Nature* 2005;436:1157–60. 10.1038/nature03891 16121181

[32] Fiegna F, Moreno-Letelier A, Bell T et al. Evolution of species interactions determines microbial community productivity in new environments. *ISME J* 2015;9:1235–45. 10.1038/ismej.2014.215 25387206 PMC4409166

[33] Fiegna F, Scheuerl T, Moreno-Letelier A et al. Saturating effects of species diversity on life-history evolution in bacteria. *Proc R Soc B Biol Sci* 2015;282:20151794. 10.1098/rspb.2015.1794 PMC4614762 26378213

[34] Rivett DW, Scheuerl T, Culbert CT et al. Resource-dependent attenuation of species interactions during bacterial succession. *ISME J* 2016;10:2259–68. 10.1038/ismej.2016.11 26894447 PMC4989303

[35] Lawrence D, Fiegna F, Behrends V et al. Species interactions alter evolutionary responses to a novel environment. *PLoS Biol* 2012;10:e1001330. 10.1371/journal.pbio.1001330 22615541 PMC3352820

[36] Zhang XX, Rainey PB. Construction and validation of a neutrally-marked strain of *Pseudomonas fluorescens* SBW25. *J Microbiol Methods* 2007;71:78–81. 10.1016/j.mimet.2007.07.001 17669526

[37] Stevenson K, McVey AF, Clark IBN et al. General calibration of microbial growth in microplate readers. *Sci Rep* 2016;6:38828. 10.1038/srep38828 27958314 PMC5153849

[38] Toming K, Kotta J, Uuemaa E et al. Predicting lake dissolved organic carbon at a global scale. *Sci Rep* 2020;10:8471. 10.1038/s41598-020-65010-3 32439876 PMC7242472

[39] Nercessian O, Noyes E, Kalyuzhnaya MG et al. Bacterial populations active in metabolism of C1 compounds in the sediment of Lake Washington, a freshwater lake. *Appl Environ Microbiol* 2005;71:6885–99. 10.1128/AEM.71.11.6885-6899.2005 16269723 PMC1287692

[40] Sereika M, Kirkegaard RH, Karst SM et al. Oxford nanopore R10.4 long-read sequencing enables the generation of near-finished bacterial genomes from pure cultures and metagenomes without short-read or reference polishing. *Nat Methods* 2022;19:823–6. 10.1038/s41592-022-01539-7 35789207 PMC9262707

[41] Rodríguez-Pérez H, Ciuffreda L, Flores C. NanoCLUST: a species-level analysis of 16S rRNA nanopore sequencing data. *Bioinformatics* 2021;37:1600–1. 10.1093/bioinformatics/btaa900 33079990

[42] De Coster W, D’Hert S, Schultz DT et al. NanoPack: visualizing and processing long-read sequencing data. *Bioinformatics* 2018;34:2666–9. 10.1093/bioinformatics/bty149 29547981 PMC6061794

[43] Li H . Minimap2: pairwise alignment for nucleotide sequences. *Bioinformatics* 2018;34:3094–100. 10.1093/bioinformatics/bty191 29750242 PMC6137996

[44] Danecek P, Bonfield JK, Liddle J et al. Twelve years of SAMtools and BCFtools. *Gigascience* 2021;10:giab008. 10.1093/gigascience/giab008 33590861 PMC7931819

[45] Latorre-Pérez A, Gimeno-Valero H, Tanner K et al. A round trip to the desert: in situ nanopore sequencing informs targeted bioprospecting. *Front Microbiol* 2021;12:768240. 10.3389/fmicb.2021.768240 34966365 PMC8710813

[46] Gasol JM, Del Giorgio PA. Using flow cytometry for counting natural planktonic bacteria and understanding the structure of planktonic bacterial communities. *Sci Mar* 2000;64:197–224. 10.3989/scimar.2000.64n2197

[47] Hawkes JA, Dittmar T, Patriarca C et al. Evaluation of the orbitrap mass spectrometer for the molecular fingerprinting analysis of natural dissolved organic matter. *Anal Chem* 2016;88:7698–704. 10.1021/acs.analchem.6b01624 27400998

[48] Schum SK, Brown LE, Mazzoleni LR. MFAssignR: molecular formula assignment software for ultrahigh resolution mass spectrometry analysis of environmental complex mixtures. *Environ Res* 2020;191:110114. 10.1016/j.envres.2020.110114 32866496

[49] R Core Team . R: A Language and Environment for Statistical Computing. Vienna, Austria: R Foundation for Statistical Computing, 2022, https://www.R-project.org/

[50] Hawkes JA, D’Andrilli J, Agar JN et al. An international laboratory comparison of dissolved organic matter composition by high resolution mass spectrometry: are we getting the same answer? *Limnol Oceanogr Methods* 2020;18:235–58. 10.1002/lom3.10364

[51] Hu A, Han L, Lu X et al. Global patterns and drivers of dissolved organic matter across earth systems: insights from H/C and O/C ratios. *Fundam Res* 2024;5:2121–32. 10.1016/j.fmre.2023.11.018 41613462 PMC12848229

[52] D’Andrilli J, Cooper WT, Foreman CM et al. An ultrahigh-resolution mass spectrometry index to estimate natural organic matter lability. *Rapid Commun Mass Spectrom* 2015;29:2385–401. 10.1002/rcm.7400 26563709 PMC4654268

[53] Koch BP, Dittmar T. From mass to structure: an aromaticity index for high-resolution mass data of natural organic matter. *Rapid Commun Mass Spectrom* 2006;20:926–32. 10.1002/rcm.2386

[54] Kim S, Kramer RW, Hatcher PG. Graphical method for analysis of ultrahigh-resolution broadband mass spectra of natural organic matter, the Van Krevelen diagram. *Anal Chem* 2003;75:5336–44. 10.1021/ac034415p 14710810

[55] Oksanen J, Simpson GL, Blanchet FG et al. Vegan: Community Ecology Package. 2022. 10.32614/CRAN.package.vegan

[56] Braga LPP, Orland C, Emilson EJS et al. Viruses direct carbon cycling in lake sediments under global change. *Proc Natl Acad Sci USA* 2022;119:e2202261119. 10.1073/pnas.2202261119 36206369 PMC9564219

[57] Menzel U. EMT: Exact Multinomial Test: Goodness-of-Fit Test for Discrete Multivariate Data. 2024. 10.32614/CRAN.package.EMT

[58] Hu A, Choi M, Tanentzap AJ et al. Ecological networks of dissolved organic matter and microorganisms under global change. *Nat Commun* 2022;13:3600. 10.1038/s41467-022-31251-1 35739132 PMC9226077

[59] Clavel J, Julliard R, Devictor V. Worldwide decline of specialist species: toward a global functional homogenization? *Front Ecol Environ* 2011;9:222–8. 10.1890/080216

[60] Taipale SJ, Brett MT, Hahn MW et al. Differing Daphnia magna assimilation efficiencies for terrestrial, bacterial, and algal carbon and fatty acids. *Ecology* 2014;95:563–76. 10.1890/13-0650.1 24669748

[61] Gushgari-Doyle S, Lui LM, Nielsen TN et al. Genotype to ecotype in niche environments: adaptation of Arthrobacter to carbon availability and environmental conditions. *ISME Commun* 2022;2:32. 10.1038/s43705-022-00113-8 37938300 PMC9723602

[62] Pepi M, Lampariello LR, Altieri R et al. Tannic acid degradation by bacterial strains *Serratia* spp. and *Pantoea* sp. isolated from olive mill waste mixtures. *Int Biodeterior Biodegradation* 2010;64:73–80. 10.1016/j.ibiod.2009.10.009

[63] Bhat TK, Singh B, Sharma OP. Microbial degradation of tannins - a current perspective. *Biodegradation* 1998;9:343–57. 10.1023/A:1008397506963 10192896

[64] Muscarella ME, Boot CM, Broeckling CD et al. Resource heterogeneity structures aquatic bacterial communities. *ISME J* 2019;13:2183–95. 10.1038/s41396-019-0427-7 31053829 PMC6775984

[65] Louca S, Doebeli M, Parfrey LW. Correcting for 16S rRNA gene copy numbers in microbiome surveys remains an unsolved problem. *Microbiome* 2018;6:41. 10.1186/s40168-018-0420-9 29482646 PMC5828423

[66] Freeman EC, Emilson EJS, Dittmar T et al. Universal microbial reworking of dissolved organic matter along environmental gradients. *Nat Commun* 2024;15:187. 10.1038/s41467-023-44431-4 38168076 PMC10762207

[67] Roth VN, Lange M, Simon C et al. Persistence of dissolved organic matter explained by molecular changes during its passage through soil. *Nat Geosci* 2019;12:755–61. 10.1038/s41561-019-0417-4

[68] Kothawala DN, Kellerman AM, Catalán N et al. Organic matter degradation across ecosystem boundaries: the need for a unified conceptualization. *Trends Ecol Evol* 2021;36:113–22. 10.1016/j.tree.2020.10.006 33168153

[69] Cavicchioli R, Ripple WJ, Timmis KN et al. Scientists’ warning to humanity: microorganisms and climate change. *Nat Rev Microbiol* 2019;17:569–86. 10.1038/s41579-019-0222-5 31213707 PMC7136171

[70] Logue JB, Stedmon CA, Kellerman AM et al. Experimental insights into the importance of aquatic bacterial community composition to the degradation of dissolved organic matter. *ISME J* 2016;10:533–45. 10.1038/ismej.2015.131 26296065 PMC4817675

[71] Catalán N, Pastor A, Borrego CM et al. The relevance of environment vs. composition on dissolved organic matter degradation in freshwaters. *Limnol Oceanogr* 2020;66:306–20. 10.1002/lno.11606

[72] Zhou L, Zhou Y, Tang X et al. Resource aromaticity affects bacterial community successions in response to different sources of dissolved organic matter. *Water Res* 2021;190:116776. 10.1016/j.watres.2020.116776 33387955

[73] D’Andrilli J, Junker JR, Smith HJ et al. DOM composition alters ecosystem function during microbial processing of isolated sources. *Biogeochemistry* 2019;142:281–98. 10.1007/s10533-018-00534-5

[74] LaBrie R, Lapierre JF, Maranger R. Contrasting patterns of labile and semilabile dissolved organic carbon from continental waters to the open ocean. *J Geophys Res Biogeosci* 2020;125:e2019JG005300. 10.1029/2019JG005300

[75] Bercovici SK, Arroyo MC, De Corte D et al. Limited utilization of extracted dissolved organic matter by prokaryotic communities from the subtropical North Atlantic. *Limnol Oceanogr* 2021;66:2509–20. 10.1002/lno.11769

