# Supplementary material for: Dissolved organic matter specialization drives temporal dynamics of simplified bacterial communities in a microcosm experiment
Source: ISME Commun. 2026 Feb 28;6(1):ycag045. doi: 10.1093/ismeco/ycag045 (PMC13011803; doi:10.1093/ismeco/ycag045)
Supplement: Sandor_ISMECOMMUN-D-25-00556R2_supplement-final_ycag045 [file sandor_ismecommun-d-25-00556r2_supplement-final_ycag045.pdf]

Supplementary Material: Dissolved organic matter specialisation drives temporal dynamics of simplified bacterial communities in a microcosm experiment

Sarah R. Sandor[1,*], Thomas Scheuerl[1,2], Jeremy A. Fonvielle[1], Caroline Kemp[1], Andrew J. Tanentzap[1,3]

1. Ecosystems and Global Change Group, Department of Plant Sciences, University of Cambridge, Cambridge, United Kingdom
2. Research Department for Limnology, University of Innsbruck, Mondsee, Austria
3. Ecosystems and Global Change Group, School of Environment, Trent University, Peterborough, Ontario, Canada

*Author for correspondence: srs69@cantab.ac.uk ; Current address: Institute for Chemistry and Biology of the Marine Environment (ICBM), Carl von Ossietzky University of Oldenburg, Carl-von-Ossietzky-Straße 9-11, 26129 Oldenburg, Germany

Supplementary Methods

DOM collection

DOM from the four Canadian freshwater samples was concentrated via reverse osmosis following collection, acidified to pH 2 and stored in Nalgene bottles at 4˚C. For the temperate pond, we collected 20 L of water in acid-rinsed Nalgene jugs and stored the water at 4˚C in the dark. Within two days, we filtered the water through pre-combusted glass microfibre filters (1.6 µm pore size, Whatman, UK) and then through 0.1 µm polyethersulfone filters (Sartorius, Germany) that were pre-rinsed with MilliQ water. We acidified the filtered water to pH 2 with HCl and stored it at 4˚C in the dark. For the subtropical river, Suwannee River natural organic

matter was purchased from the International Humic Substances Society (batch 2R101N). To prepare a stock concentrate, we dissolved the freeze-dried powder in MilliQ water in a pre-combusted glass bottle, acidified it to pH 2 with HCl and stored it at 4˚C. To create the beech leaf source, we collected freshly fallen beech leaves in Cambridge, UK and dried them overnight at 60˚C. We combined 50 g of dried leaves with 500 mL of deionized water in a pre-combusted glass bottle and autoclaved the mixture. After cooling overnight, we filtered the leachate through a cellulose filter (11 µm pore size, Whatman, USA) into a pre-combusted glass bottle and autoclaved the leachate again, then stored the leachate in the dark at 4˚C.

DOM extraction and concentration for media preparation

To concentrate and extract the DOM from each source for the bacterial media, we first measured the dissolved organic carbon (DOC) concentrations using a TOC-L analyzer (Shimadzu, Japan). We then diluted each DOM substrate in MilliQ water to approximately 20 mg C L^{-1} and acidified the dilutions to pH 2 with HCl. Approximately 2.4 mg of carbon was passed through activated styrene-divinylbenzene PPL cartridges (1 g, Agilent, USA) with gravity. Cartridges were first cleaned by soaking each in HPLC-grade methanol (Merck, USA) overnight and activated by rinsing them MilliQ water at pH 2 immediately before loading each sample. After collecting the carbon on the cartridges, we desalted them by passing five cartridge volumes of MilliQ water at pH 2 through each one and drying the cartridges by centrifugation at 3,200 × *g* for 30 minutes. We eluted the DOM with approximately 9 mL of HPLC-grade methanol into pre-combusted (5 hours at 500˚C) amber glass vials and stored the extracts at -20˚C.

Prior to creating the bacterial media, we dried the resulting extracts in pre-combusted glass tubes in a speed vacuum overnight to yield approximately 15 mg C from each DOM source. We re-dissolved the extracted DOM in MilliQ water and sonicated the samples in a

water bath, filter-sterilized the concentrates through pre-rinsed 0.1µm PES filters (Sartorius, Germany), and stored the concentrates in pre-combusted glass bottles at 4˚C until the media was prepared.

Bacterial media and community preparation

All bacterial media was prepared using a modified minimal M9 media recipe with either glycerol (for the lab acclimation from frozen culture) or each DOM source added as the sole carbon source. Standard M9 media contains a higher salt concentration (11.5 g L^{-1}) than observed in freshwaters. Therefore, to create more ecologically relevant media, we reduced the amount of all salts added to the media by 75%. This concentration of salts was the minimum required to maintain the pH at ca. 7.3, which is the optimum pH for the species to grow [1]. All components of the M9 media were either autoclaved (M9 salts, MilliQ) or filter-sterilized ($MgSO_4$, $CaCl_2$) through a 0.22µm syringe filter (Sartorius, Germany) that was pre-rinsed with autoclaved MilliQ water to remove any residual carbon. Reagents and the bacterial cultures were prepared in pre-combusted (5 hours at 500˚C) glass bottles. The final six-species community was assembled in a pre-combusted glass bottle and mixed thoroughly prior to inoculating each experimental bottle.

DNA extractions

Sterivex filter cartridges were opened under sterile conditions and filters placed in sterile Eppendorf tubes with sterile silica (3 mm diameter) and zirconia (0.7 mm and 0.1 mm diameter) beads, 0.6 mL of 10% m/v cetrimonium bromide in 1.6 M NaCl, and 60 µL each of 10% sodium dodecyl sulfate and 10% N-Lauroylsarcosin. We vortexed the tubes at 3,200 rpm for 10 minutes to detach the bacterial cells from the filter and disrupt cell membranes, added 0.6 mL of 25:24:1 phenol-chloroform-isoamyl alcohol (pH 7) and vortexed the tubes for

another 10 minutes. To separate the DNA from the cellular and filter debris, we centrifuged the tubes at 16, 000 × *g* for 10 minutes at 4˚C. The aqueous upper phase was transferred to a clean Eppendorf tube with 24:1 chloroform-isoamyl alcohol and centrifuged at 16, 000 × *g* for another 10 minutes at 4˚C. We transferred the aqueous upper phase again to a new Eppendorf tube and added 1.1 mL of 30% (m/v) polyethylene glycol in 1.6 M NaCl to precipitate the DNA and incubated the samples overnight at 4˚C. The next day, we centrifuged the samples at 17, 000 × *g* for one hour at 4˚C. After removing the supernatant, we washed the DNA pellet with ice-cold 70% ethanol and centrifuged at 17, 000 × *g* for 15 minutes. After the ethanol was removed, we placed the tubes on a 37˚C heating block to dry the DNA pellet. We then dissolved the DNA in 50 µL of nuclease-free water. Final DNA concentrations were quantified using a Qubit fluorometer (Thermo Fisher Scientific, USA).

Sequencing library preparation

We diluted all DNA samples to a concentration of 1 ng DNA μL^{-1} and combined 10 µL of DNA with 14 µL of nuclease free water, 25µL of LongAmp Taq 2× Master Mix (New England Biolabs, USA) and 1 µL of barcode. After mixing by pipetting, we ran a PCR on a SimpliAmp thermocycler (Applied Biosystems, USA), with an initial denaturation step at 95˚C for 1 minute followed by 25 cycles of a 95˚C denaturation step for 20 seconds, an annealing step at 55˚C for 30 seconds, an extension step at 65˚C for 2 minutes, and a final extension at 65˚C for 5 minutes. The 16S PCR products were purified by adding 30 µL of Illumina beads (Illumina, USA), centrifuging for 5 minutes and pelleting the beads containing the PCR fragments using a magnetic stand. After removing the supernatant, we washed the beads twice with 200 µL of 70% ethanol and air dried them before resuspension in 10 µL of TE buffer (LGC Biosearch Technologies, UK). We then incubated the tubes for 2 minutes at room temperature to detach the PCR fragments from the beads, and pelleted again with the magnet

to remove the supernatant containing the DNA. All final PCR products were quantified using a Qubit fluorometer (Thermo Fisher Scientific, USA). Each sequencing library eventually contained up to 12 samples, each with an individual barcode, with a total DNA quantity of 50-100 ng. We incubated the DNA for each library (10 µL) with 1 µL of Rapid Adapter for 5 minutes at room temperature and combined 5 µL of each library with well-mixed Sequencing Buffer II (15 µL) and Loading Beads II (10 µL) before loading onto the flongle flow cells.

DNA extraction of microbial community standard

We prepared the positive control sample for extraction by replicating how each of the experimental samples were processed. We combined 75 µL of thawed ZymoBIOMICS® Microbial Community Standard II (Log Distribution) (Zymo Research, USA) with 100 mL of M9 media (25% salts, no carbon substrate), and passed it through a Sterivex filter (Millipore, USA). The filter was frozen at -80˚C until further processing. We then extracted DNA from the filter and amplified and sequenced the 16S gene following the same protocol as the experimental samples. We processed and sequenced the microbial standard four times.

16S database of study species

To create the database, we extracted the contig containing the 16S gene sequence from the whole genome assemblies of the five beech tree species downloaded from ENA project PRJEB34793 [1]: *Arthrobacter* sp. (accession GCA_902706295; contig 46), *Pantoea* sp. (accession GCA_902706165; contig 18), *Pseudomonas fragi* (accession GCA_902706265; contig 34), *Raoultella* sp. (accession GCA_902706135; contig 33) and *Sphingobacterium* sp. (accession GCA_902706145; contig 19). We compiled the contig sequences into a multi-FASTA file and added the complete 16S sequence of *Pseudomonas fluorescens* SBW25 [2] from the most recent whole genome entry on NCBI (accession number OV986001.1).

Flow cytometry settings

Each sample was stained with SYBR Green I (Thermo Fisher Scientific, USA) at a final concentration of 1× overnight in the dark at 4˚C. The next day, we briefly vortexed the samples to mix them, then counted 400µL of each sample on an Attune NxT flow cytometer (Thermo Fisher Scientific, USA) with a 530/30nm filter and forward scatter and side-scatter thresholds set to 15K and 10K, respectively. We also ran an unstained aliquot of three bacterial samples from each DOM source to define the gating within the Attune Cytometric Software (Fig. S8). Prior to analyzing the cell count data, all values were corrected to remove background signal identified in the controls.

DOM extractions

To characterise the organic matter composition of each sample collected during the incubation, approximately 0.24 mg of carbon was passed through a 100 mg styrene-divinylbenzene PPL cartridge (Agilent, USA) that was previously cleaned and activated as described in *DOM extraction and concentration for media preparation*. We then aliquot ca. 48 µg C from each extracted sample into a clean Lo-bind Eppendorf tube and dried the samples in a speed vacuum for 2 hours at room temperature. The dried extracts were then dissolved in 120µL of solvent containing MilliQ water (95%), acetonitrile (5%), and formic acid (0.2%). The final DOM extracts were eluted with approximately 3 mL of HPLC-grade methanol into pre-combusted amber glass vials and stored at -20˚C until mass spectrometry analysis.

Liquid chromatography – high-resolution mass spectrometry settings

We separated analytes using reversed phase chromatography and a Acquity BEH C18 column (Waters Corporation, USA) using 0.1% formic acid in water as mobile phase A and

0.1% formic acid in 80% acetonitrile as mobile phase B. Separation occurred over 9 minutes using the following gradient: 0% mobile phase B for 0.5 minutes, up to 80% mobile phase B at 6 minutes, 80% B from 6 minutes to 7.5 minutes, down to 0% B at 8 minutes, followed by 1 minute of equilibration (0% B). All reagents were LC-MS grade (LiChrosolv; Merck, UK). The electrospray ionization source was set to −3 kV, 100 °C, and the Orbitrap was set to collect data at a resolution setting of 70000. We exported the raw spectra and converted them into centroided mzXML format [3] using ReAdW version 4.3.1 (http://tools.proteomecenter.org/wiki/index.php?title=Software:ReAdW).

Formula assignment pipeline

Formulae were assigned to each mass list individually. We first summed all scans from each sample into one integrated mass list using the *MSnbase* package [4] in R and discarded regions before 1.5 min as they did not contain any data. We examined each raw spectrum and removed obvious contaminants. Then, we estimated noise using the Kendrick Mass Defect method, implemented with the 'KMDNoise' function of *MFAssignR* [5] and removed all compounds with an intensity lower than two times the estimated noise. We performed internal calibration using a linear model and a list of 76 internal calibrants provided by *Formularity* software [6]. Briefly, we used a 2 ppm threshold to determine which calibrants were present in a given spectrum and modeled the theoretical versus the observed masses of these calibrants to obtain calibrated masses. We assigned formulae on the resulting mass list in two rounds. In the first round, we ran the 'MFAssign' function in *MFAssignR* for CHO compounds only. Then, we ran the function 'MFAssign' a second and allowed up to four nitrogen (N) and up to two sulfur (S) atoms per formulas and an error of 3 ppm. All formulae with N or S were retained only if they were part of a CH2 homologous series [7]. In addition, we removed formulae that did not match any of the following criteria: $H \leq 4C$; $O < 1.2C$; $N \leq C+1$; $S \leq C+1$ [7]; number

of double bond equivalent and the number of oxygen atoms was lower than 10 [8]. We used the resulting mass list to refine molecular formulae attribution in a second round. Within the second round, we recalibrated masses using a list of recalcitrant series obtained from the 'Recal' function of *MFassignR* and assigned formulae to the recalibrated mass list. We used ^{13}C isotopes to confirm formulae attribution and applied the same filters as in the first round. We modelled the error of all formulae as a function of their mass using quantile regression and the package *quantreg* [9]. We removed formulae with mass errors that were not within 1.5 times the interquartile range of the obtained model as those were unlikely [7]. Finally, we manually removed 29 remaining obvious contaminants observed across the entire dataset and 32 peaks with m/z values matching to a list of known contaminants (Table S8). Assigned formulae from two replicates of the Suwannee River natural organic matter reference material (batch 2R101N) run alongside our samples fell within expected guidelines from an inter-laboratory standard comparison [10] for all metrics except the O/C ratio (H/C = 1.04 and 1.06; O/C = 0.64 and ; m/z = 384.4 and 378.9; AI_{mod} = 0.33 and 0.32), thereby validating our analysis.

We classified each molecular formula into the following putative compound classes based on their H/C and O/C ratios: condensed aromatic-like (O/C $\leq$ 0.67 and 0.2 $\leq$H/C < 0.7), unsaturated hydrocarbon-like (O/C $\leq$ 0.1 and 0.7 $\leq$ H/C < 1.5), lignin-like (0.1 < O/C $\leq$ 0.67 and 0.7 $\leq$ H/C < 1.5), tannin-like (0.67 $\leq$ O/C $\leq$ 1.2 and 0.5 $\leq$ H/C < 1.5), lipid-like (O/C $\leq$ 0.3 and 1.5 $\leq$ H/C $\leq$ 2), protein-like (0.3 < O/C $\leq$ 0.55 and 1.5 $\leq$ H/C $\leq$ 2.2), amino sugar-like (0.55 < O/C $\leq$ 0.67 and 1.5 $\leq$ H/C $\leq$ 2.2) and carbohydrate-like (0.67 < O/C $\leq$ 1.2 and 1.5 $\leq$ H/C $\leq$ 2).

Statistical analyses

For the distance-based redundancy analysis, we set the Bray-Curtis dissimilarities among bacterial communities as the response variable, with bulk DOM metrics and summed

relative intensities of each compound class as explanatory variables. Statistical significance was tested using the 'anova.cca' function with 999 randomised permutations.

To test how bacteria and DOM changed over time, we first fitted a separate PERMANOVA to bacterial community composition on each DOM source with time as a continuous predictor. For sources where the bacterial community composition changed with time, we combined those datasets together and fitted a second PERMANOVA with an interaction between DOM source and time to test if the temporal changes differed among sources.

Supplementary Figures and Tables

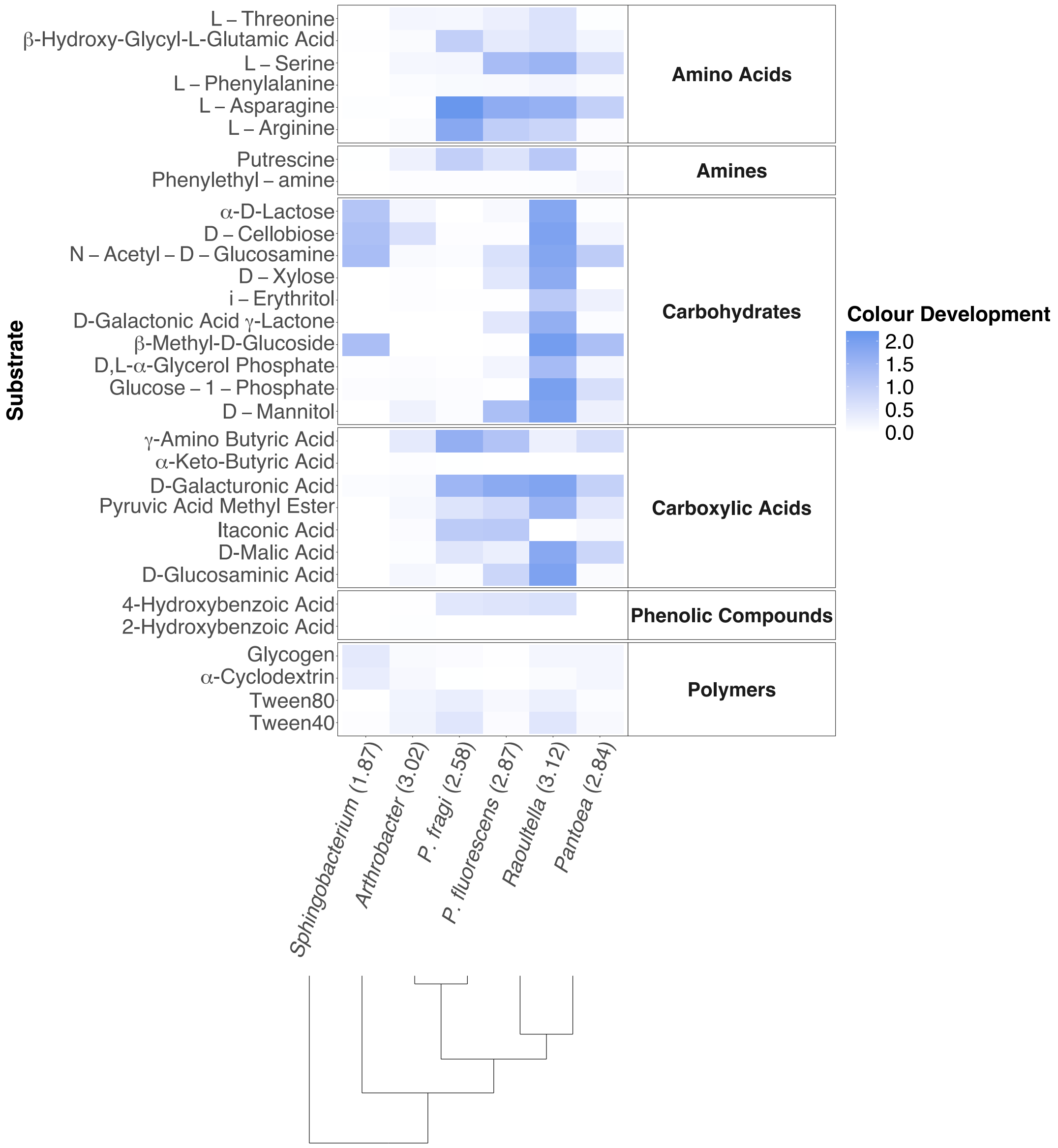

Figure S1 | Resource use of each species. Each species was grown in monoculture in R2A media on 31 different organic substrates in a Biolog EcoPlate. Absorbance was measured at 590nm and 750nm after 7 days of growth in the dark at 22˚C. Colour development for each substrate was calculated by first subtracting absorbance at 750nm from that at 590nm to correct for turbidity. We then blank-corrected each value by subtracting the control (no substrate) well. To account for differences in the starting cell densities of each species, we further subtracted values measured immediately after inoculating the plates [11]. Darker colours indicate greater consumption of the substrate. The dendrogram shows the phylogenetic relationships among the six species based on partial 16S rRNA gene sequences. Numbers in brackets indicate the diversity of substrates consumed by each species, measured as the Shannon diversity index.

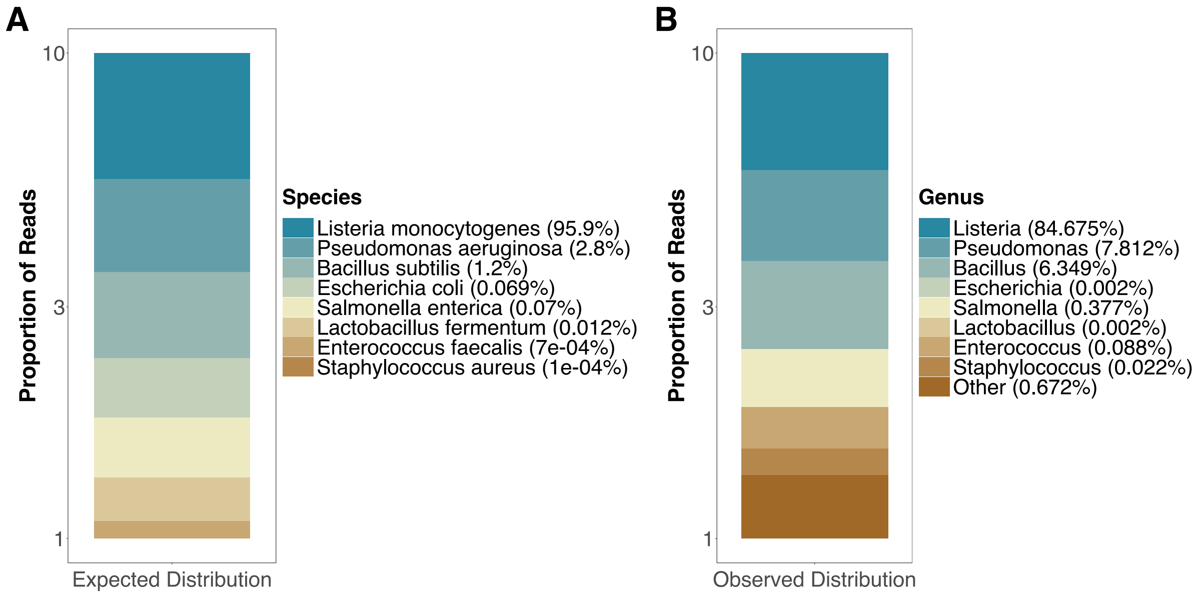

Figure S2 | The observed composition of the positive ZymoBIOMICS® microbial community control was similar to the expected theoretical distribution. The **(A)** expected relative abundance of each species and **(B)** the observed proportion of reads that mapped to each of the eight genera present in the community standard. An additional 0.67% of reads in **(B)** did not map to any of the eight genera and are labelled as 'Other'. There was no statistically significant difference between the expected and observed distributions (paired Wilcoxon signed rank test: V = 32, p = 0.301).

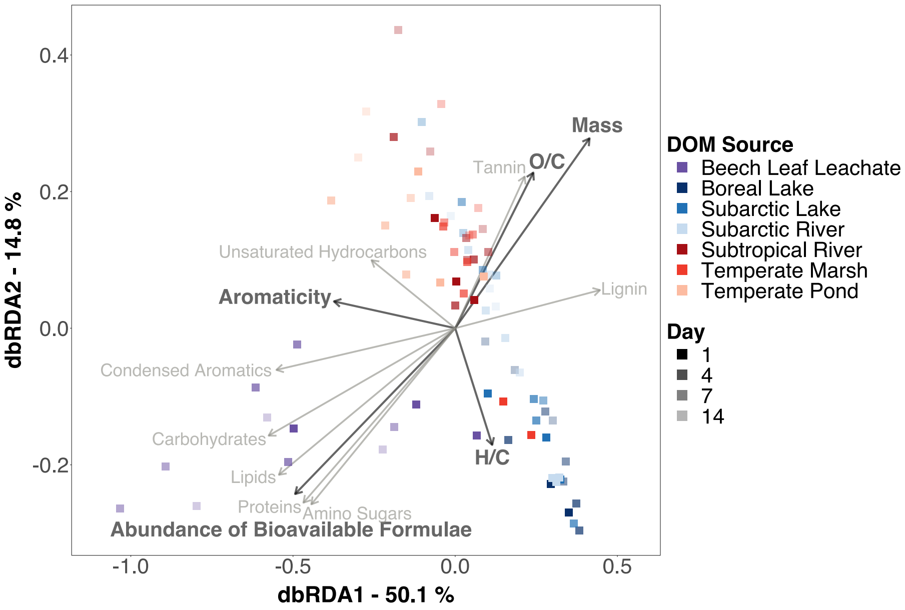

Figure S3 | DOM composition explained variation in bacterial community composition. We conducted a distance based redundancy analysis (dbRDA) of the bacterial communities across the time series (n = 80) based on Bray-Curtis dissimilarities to quantify the variation in community composition that is explained by the molecular composition of the DOM. Each point is a sample, with the distance between points related to sample similarity. Grey arrows show the explanatory variables from the dbRDA: average intensity-weighted H/C ratio, O/C ratio, aromaticity (modified aromaticity index), mass (mass to charge ratio), abundance of bioavailable formulae (relative intensity of formulae with H/C ratio >1.5) and relative abundance of formulae from different compound classes. Explanatory variables from the dbRDA are shows Percentages indicate the variation explained by each axis.

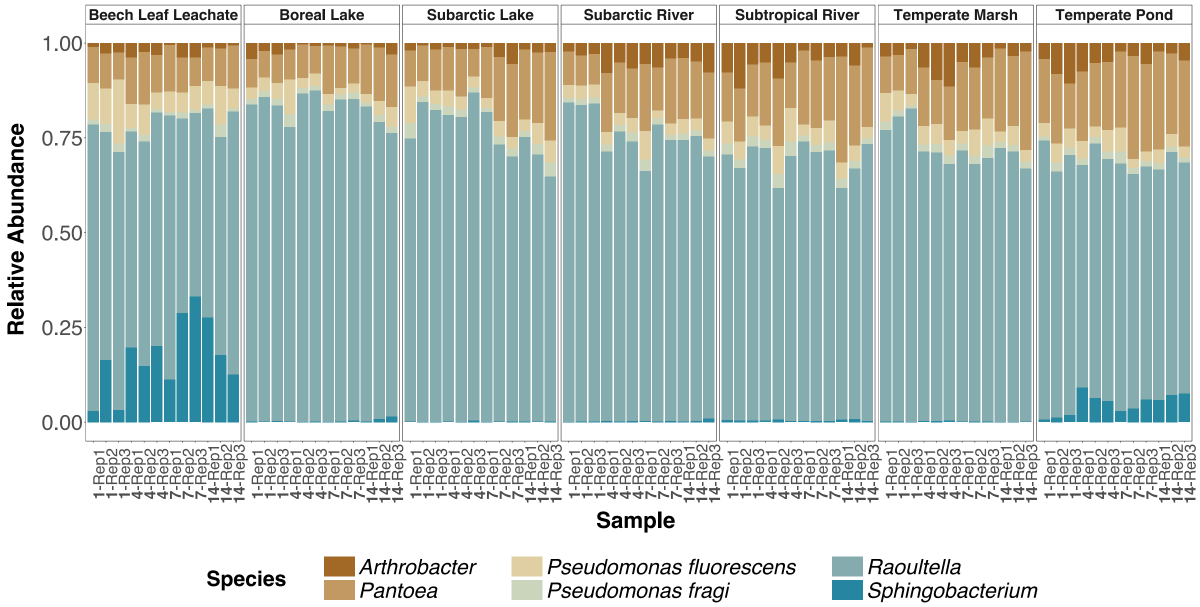

Figure S4 | *Raoutella* dominated the six-species communities despite differences in community composition between DOM sources. The relative abundance of each species across the time series is shown by the coloured bars. Samples are arranged from day 1 to 14 on the x-axis. The DOM source panels are arranged based on the PCoA groupings (Fig. 2) with the labile sources on the left (beech leaf leachate, boreal lake, subarctic lake and subarctic river) and the recalcitrant sources on the right (subtropical river, temperate marsh and temperate pond).

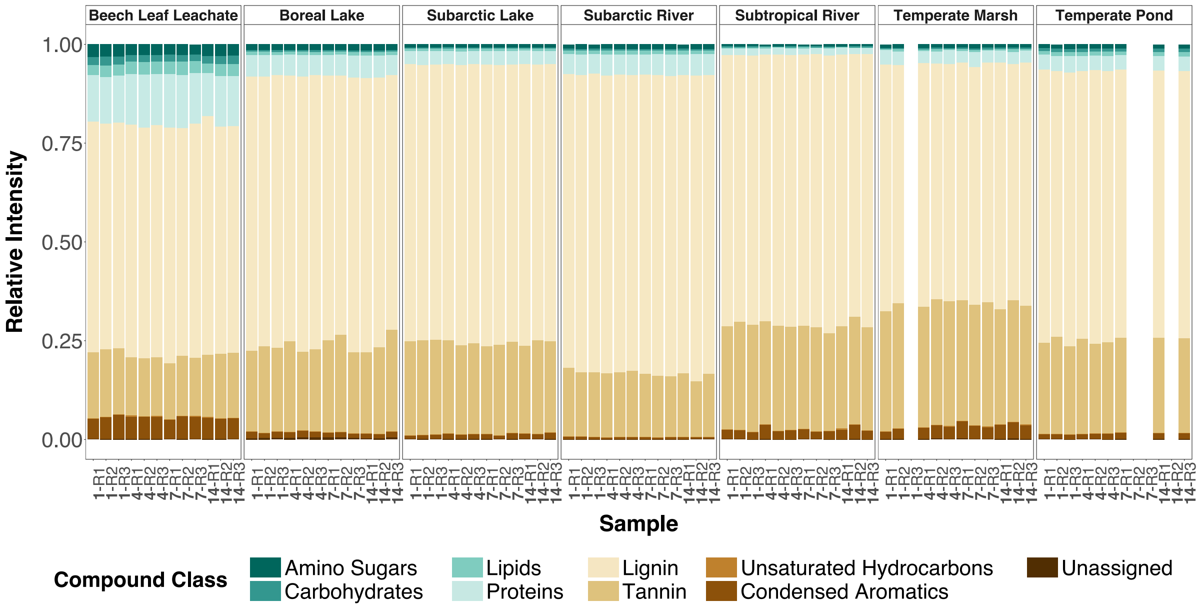

Figure S5 | DOM sources were dominated by lignin- and tannin-like formulae. The summed relative intensity of different putative compound classes are shown in the coloured bars. The compound classes with a higher bioavailability (H/C ratio > 1.5) are in shades of green while the less bioavailable classes are in shades of brown. Samples are arranged from day 1 to 14 on the x-axis. The DOM source panels are arranged based on the PCoA groupings (Fig. 1) with the labile sources on the left (beech leaf leachate, boreal lake, subarctic lake and subarctic river) and the recalcitrant sources on the right (subtropical river, temperate marsh, temperate pond).

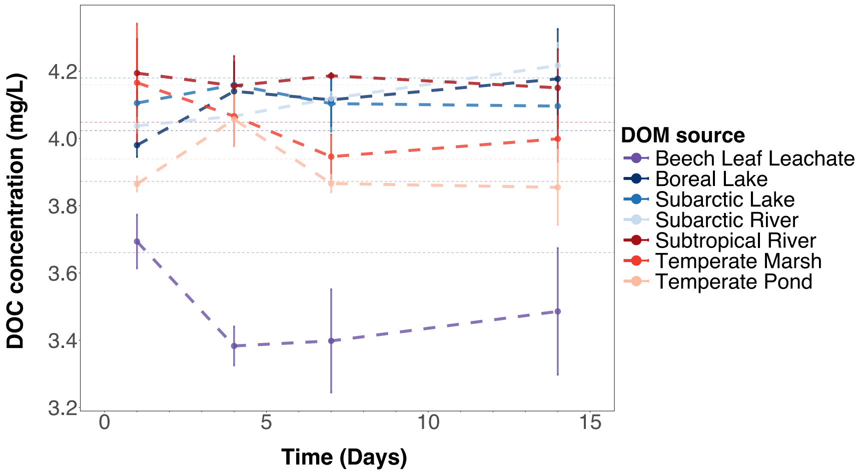

Figure S6 | DOC concentrations were constant over time. Each point is the average ± standard error DOC concentration across the three replicate bottles at every time point. The dashed horizonal lines in are the average DOC concentrations of each source at the start of the experiment. None of the DOC concentrations changed over time (ANOVA: $F_{1,\ 54.2} = 0.07$, p = 0.79).

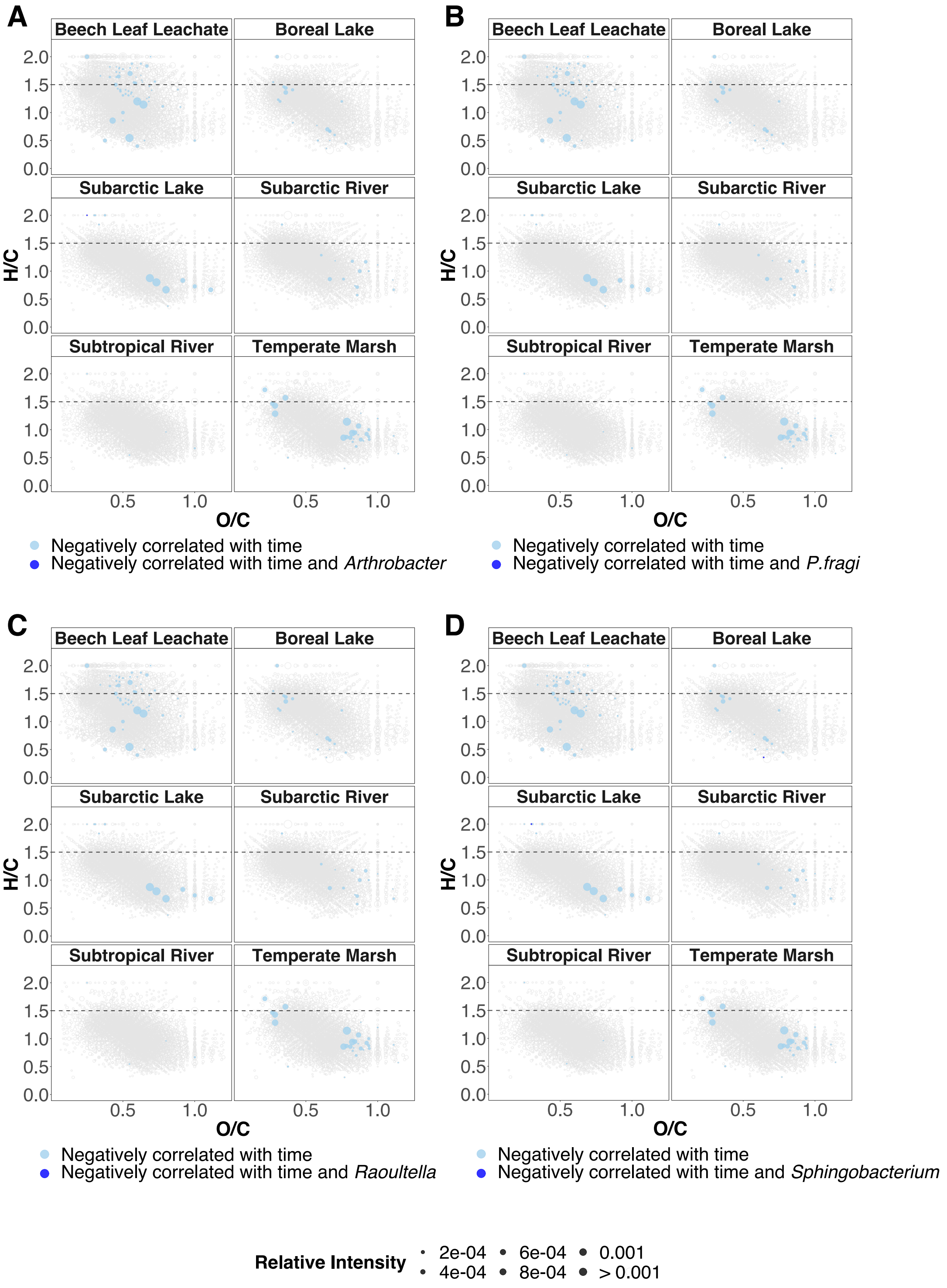

Figure S7 | van Krevelen diagrams of resource use of *Arthrobacter, P. fragi, Raoultella* and *Sphingobacterium*. Individual molecular formulae are represented by grey circles sized based on their relative intensity at day 1 averaged across three replicates per DOM source. Coloured points show formulae that were either strongly negatively correlated with time but not the focal species (i.e. light blue) or negatively correlated with both time and the focal species (dark blue).

Negative correlations were defined as the strongest 2.5% of correlations, corresponding to $\rho \leq -0.706$ in the dataset of species-by-formulae correlations and $\rho \leq -0.806$ in the dataset of formulae-by-time correlations. The dashed line at H/C = 1.5 indicates the biolability boundary.

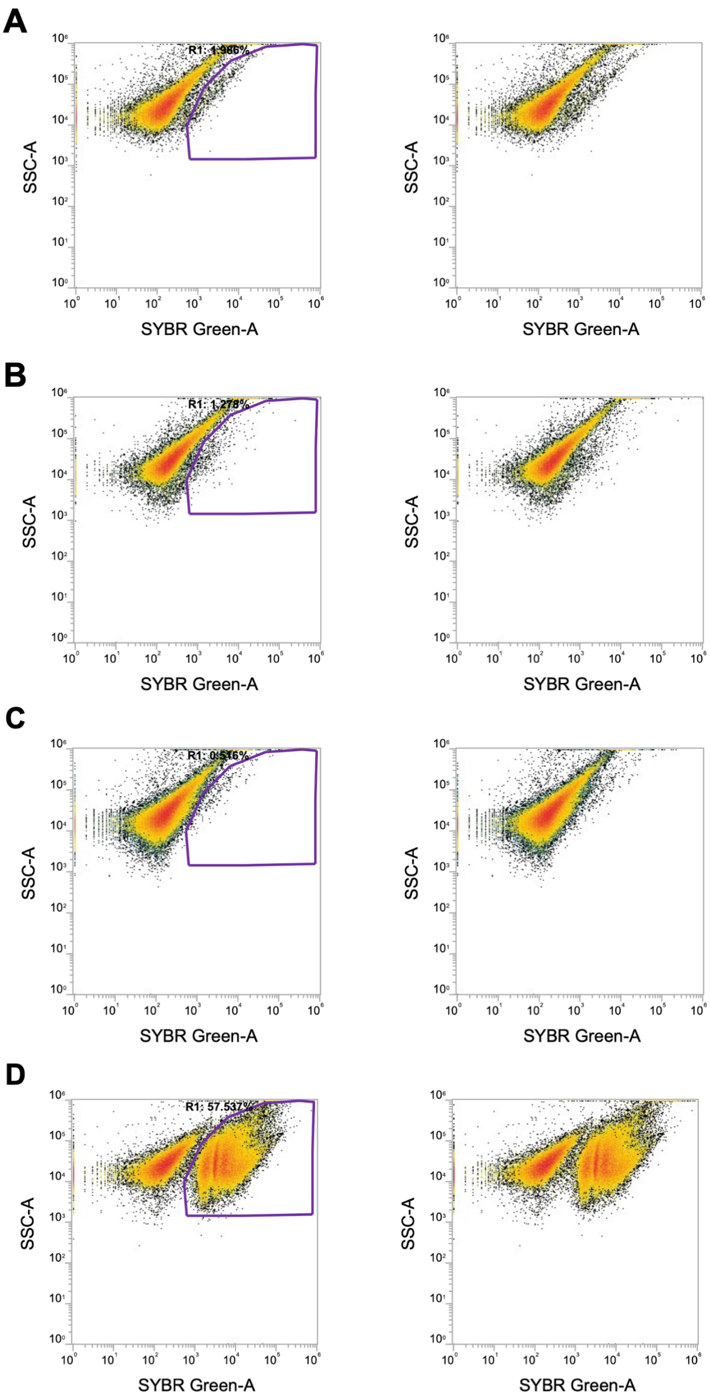

Figure S8 | Example of the flow cytometry gating. Flow cytometry results for beech leaf leachate samples at day 1. **(A)** No bacterial control not stained with SYBR-Green. **(B)** No bacteria control stained with SYBR-Green. **(C)** Bacterial sample, replicate one, not stained with SYBR-Green. **(D)** Bacterial sample, replicate one, stained with SYBR-Green. Plots **A-C** were used to define the background signal.

Table S1 | Bacterial community composition differed between DOM sources and changed similarly over time. We fitted a PERMANOVA to the bacterial community composition data for each DOM source to test if the compositions changed over time. For the communities that changed with time, we then fit another PERMANOVA to the composition data of these samples to test if the changes differed among sources. The table summarizes the F statistic and p-value for each PERMANOVA. The DOM sources are coloured as in Fig. 1 based on DOM composition. Bolded rows had statistically significant changes in community composition over time.

**DOM Source**	**F**	**R2**	***p***
	Individual sources		
Beech Leaf Leachate	1.10	0.11	0.325
**Boreal Lake**	**5.43**	**0.38**	**0.009**
**Subarctic Lake**	**8.14**	**0.49**	**0.012**
**Subarctic River**	**4.65**	**0.33**	**0.045**
**Subtropical River**	**7.35**	**0.38**	**0.001**
**Temperate Marsh**	**6.39**	**0.41**	**0.001**
**Temperate Pond**	**5.87**	**0.41**	**0.006**
	All sources that varied with time		
Source × Time	1.68	0.04	0.174

Table S2 | Statistical summary of correlation between environmental variables and principal coordinates (PCoA) analysis. Bulk DOM metrics, relative abundances of putative compound classes and relative abundances of each bacterial species of each sample were correlated with the axes of the DOM composition and bacterial community composition PCoA ordinations. For each of the two ordinations, the squared correlation coefficient (r^2) and p-value (*p*) based on 999 permutations are show for every variable tested. H/C, O/C, Mass and Aromaticity indicate the average intensity weighted H/C ratio, O/C ratio, m/z value and AI_{mod}, respectively. Abundance of bioavailable formulae indicates the summed relative intensity of all bioavailable formulae (i.e. H/C > 1.5). * indicates a statistically significant correlation between the variable and the ordination.

	**DOM Composition PCoA**		**Bacterial Community PCoA**	
	**r^2**	***p***	**r^2**	***p***
**Bulk DOM Metrics**				
H/C	0.8285	0.001*	0.0960	0.019*
O/C	0.8020	0.001*	0.3064	0.001*
Mass	0.9616	0.001*	0.6633	0.001*
Aromaticity	0.8466	0.001*	0.2012	0.001*
Abundance of Bioavailable Formulae	0.9758	0.001*	0.6975	0.001*
**Relative abundances of each putative compound class**				
Amino Sugars	0.9463	0.001*	0.6598	0.001*
Carbohydrates	0.9025	0.001*	0.6835	0.001*
Lipids	0.9404	0.001*	0.7219	0.001*
Proteins	0.9756	0.001*	0.6827	0.001*
Lignin	0.7911	0.001*	0.3535	0.001*
Tannin	0.7526	0.001*	0.2722	0.001*
Condensed Aromatics	0.9321	0.001*	0.5036	0.001*
Unsaturated Hydrocarbon	0.0357	0.236	0.1123	0.016*
Unassigned	0.0832	0.041 *	0.2248	0.001*
**Relative abundance of each bacterial species**				
Arthrobacter	0.1768	0.001*	0.2381	0.001*
Pantoea	0.3090	0.001*	0.8698	0.001*
P. fluorescens	0.3019	0.001*	0.1998	0.003*
P. fragi	0.1219	0.014*	0.0754	0.050*
Raoultella	0.4401	0.001*	0.9825	0.001*
Sphingobacterium	0.6870	0.001*	0.9465	0.001*

Table S3 | Changes in species abundances over time. We fitted generalized linear models with a quasibinomial binomial error structure and time as a predictor to the relative abundance of each species changed on each DOM source. Values show the mean (± standard error) for the intercept (top set of values in each cell) and effect of time (bottom set of values). Statistically significant values are indicated with *** $p < 0.001$, ** $p < 0.01$ and * $p < 0.05$. Bolded values indicate that the relative abundance changed over time. The DOM sources are coloured as in Fig. 1 with the labile sources in purple and blue and the recalcitrant sources in red.

DOM Source	*Arthrobacter*	*Pantoea*	*P. fluorescens*	*P. fragi*	*Raoultella*	*Sphingobacterium*
Beech Leaf Leachate	-3.81 (0.42)***<br>-0.04 (0.05)	-2.11 (0.15)***<br>0.01 (0.02)	-2.30 (0.31)***<br>-0.06 (0.04)	-4.46 (0.29)***<br>-0.02 (0.03)	0.75 (0.21)**<br>-0.02 (0.02)	-2.22 (0.46)**<br>0.07 (0.05)
Boreal Lake	-3.75 (0.46)***<br>-0.05 (0.05)	-2.54 (0.09)***<br>**0.05 (0.01)*** **	-3.05 (0.35)***<br>-0.01 (0.04)	-3.83 (0.24)***<br>-0.01 (0.02)	1.69 (0.16)***<br>-0.03 (0.01)	-8.12 (0.58)***<br>**0.16 (0.04)** **
Subarctic Lake	-4.55 (0.46)***<br>0.05 (0.04)	-2.38 (0.17)***<br>**0.07 (0.02)** **	-2.67 (0.28)***<br>-0.05 (0.03)	-3.66 (0.31)***<br>-0.02 (0.03)	1.55 (0.23)***<br>-0.04 (0.02)	-7.04 (0.46)***<br>0.04 (0.04)
Subarctic River	-3.10 (0.27)***<br>0.02 (0.02)	-2.05 (0.16)***<br>**0.04 (0.01)* **	-3.03 (0.22)***<br>-0.003 (0.02)	-3.87 (0.19)***<br>0.001 (0.02)	1.25 (0.18)***<br>-0.03 (0.02)	-6.85 (0.32)***<br>**0.09 (0.02)** **
Subtropical River	-2.51 (0.18)***<br>**-0.09 (0.02)** **	-1.82 (0.08)***<br>**0.05 (0.01)*** **	-2.87 (0.20)***<br>-0.04 (0.02)	-3.55 (0.19)***<br>-0.03 (0.02)	0.85 (0.11)***<br>-0.01 (0.01)	-5.47 (0.21)<br>0.02 (0.02)
Temperate Marsh	-3.01 (0.43)***<br>-0.04 (0.05)	-2.15 (0.08)***<br>**0.07 (0.01)*** **	-2.71 (0.19)***<br>-0.03 (0.02)	-3.88 (0.19)***<br>-0.003 (0.02)	1.19 (0.14)***<br>-0.03 (0.01)	-6.15 (0.30)***<br>-0.05 (0.04)
Temperate Pond	-2.69 (0.23)***<br>**-0.09 (0.02)** **	-1.65 (0.16)***<br>**0.04 (0.01)* **	-2.85 (0.20)***<br>-0.04 (0.02)	-3.80 (0.21)***<br>-0.02 (0.02)	0.74 (0.11)***<br>-0.02 (0.01)	-3.74 (0.51)***<br>**0.09 (0.04)* **

Table S4 | Molecular composition changed over time in four of the seven sources. We fitted a PERMANOVA to the molecular composition data for each DOM source to test if the molecular composition changed over time. For the sources that changed with time, we then fit another PERMANOVA to the composition data of these sources to test if the changes differed among sources. The table summarizes the F statistic and *p*-value for each PERMANOVA. The DOM sources are coloured as in Fig. 1 based on DOM composition. Bolded rows had statistically significant changes in molecular composition over time.

**DOM source**	**F**	**R^2**	***p***
	Individual sources		
**Beech Leaf Leachate**	**3.76**	**0.28**	**0.007**
Boreal Lake	1.69	0.16	0.102
**Subarctic Lake**	**2.34**	**0.18**	**0.018**
**Subarctic River**	**2.30**	**0.18**	**0.034**
Subtropical River	1.36	0.13	0.244
**Temperate Marsh**	**3.42**	**0.31**	**0.043**
**Temperate Pond**	**2.74**	**0.28**	**0.048**
	All sources that varied with time		
**Source × Time**	**3.37**	**0.02**	**0.039**

Table S5 | The relative abundance of formulae from each putative compound class do not change over time. We tested if the average intensity-weighted relative abundance of formulae from each putative compound class changed over time using linear models (logit-transformed data with time as a predictor) for each DOM source. Values show the mean (± standard error) for the intercept (top set of values in each cell) and effect of time (bottom set of values) with the percent change in the relative abundance between day 1 and day 14. Statistical significance is indicated with *** at $p < 0.001$, ** at $p < 0.01$ and * at $p < 0.05$. Bolded and shaded value indicate compound classes that changed significantly over time with a greater than 10% change between day 1 and day 14.

Compound Class	Beech Leaf Leachate	Boreal Lake	Subarctic Lake	Subarctic River	Subtropical River	Temperate Marsh	Temperate Pond
Amino Sugars	-3.48 (0.05) ***<br>-0.002 (0.01)	-4.21 (0.02) ***<br>0.003 (0.002)	-4.79 (0.01) ***<br>-0.001 (0.001)	-4.45 (0.01) ***<br>0.000 (0.000)	-5.26 (0.04) ***<br>0.002 (0.003)	-4.63 (0.07) ***<br>-0.001 (0.007)	-4.53 (0.04) ***<br>0.003 (0.004)
Carbohydrates	-4.13 (0.09) ***<br>-0.000 (0.01)	-5.52 (0.04) ***<br>0.005 (0.004)	-5.95 (0.03) ***<br>-0.000 (0.003)	-5.44 (0.02) ***<br>-0.002 (0.002)	-6.02 (0.16) ***<br>-0.014 (0.015)	-5.92 (0.18) ***<br>0.029 (0.017)	-4.93 (0.07) ***<br>0.007 (0.007)
Lipids	-3.54 (0.06) ***<br>-0.001 (0.005)	-4.75 (0.04) ***<br>-0.004 (0.004)	-5.07 (0.01) ***<br>-0.000 (0.001)	-4.60 (0.02) ***<br>-0.001 (0.001)	**-5.76 (0.17) ***<br>-0.050 (0.016) *<br>-50.03%**	**-5.78 (0.08) ***<br>0.012 (0.007) *<br>+18.2%**	-4.64 (0.05) ***<br>0.008 (0.005)
Proteins	-1.96 (0.05) ***<br>-0.000 (0.004)	-2.88 (0.03) ***<br>0.002 (0.002)	-3.37 (0.01) ***<br>0.000 (0.001)	-2.94 (0.02) ***<br>0.003 (0.002)	-4.11 (0.04) ***<br>-0.005 (0.004)	-3.37 (0.06) ***<br>-0.001 (0.006)	-3.21 (0.02) ***<br>-0.006 (0.002)*<br>-6.8%
Condensed Aromatics	-2.84 (0.04) ***<br>-0.010 (0.003)	-4.16 (0.12) ***<br>-0.016 (0.012)	**-4.60 (0.07) ***<br>0.023 (0.007) *<br>+40.5%**	-5.27 (0.09) ***<br>-0.003 (0.009)	-3.67 (0.14) ***<br>0.014 (0.013)	**-3.68 (0.13) ***<br>0.034 (0.013) *<br>+70.2%**	-4.32 (0.07) ***<br>0.011 (0.008)
Lignin	0.40 (0.02) ***<br>0.001 (0.002)	0.82 (0.05) ***<br>-0.005 (0.005)	0.90 (0.02) ***<br>0.002 (0.002)	1.08 (0.02) ***<br>0.005 (0.001) *<br>+ 1.59%	0.79 (0.03) ***<br>-0.000 (0.003)	0.48 (0.02) ***<br>0.003 (0.002)	0.80 (0.03) ***<br>-0.004 (0.003)

Tannin	-1.78 (0.05) ***<br>0.000 (0.005)	-1.35 (0.07) ***<br>0.008 (0.007)	-1.22 (0.02)<br>-0.004 (0.002)	-1.58 (0.03) ***<br>-0.008 (0.003) *<br>-8.43%	-1.06 (0.02) ***<br>-0.000 (0.002)	-0.82 (0.01) ***<br>-0.008 (0.001) ***<br>-6.0%	-1.21 (0.03) ***<br>0.005 (0.003)
Unsaturated Hydrocarbons	-6.79 (0.38) ***<br>-0.005 (0.036)	**-7.38 (0.04) ***<br>0.009 (0.004) *<br>+13.1%**	-7.12 (0.05)<br>0.008 (0.005)	-6.98 (0.02) ***<br>0.007 (0.002) **<br>+9.3%	-6.97 (0.39) ***<br>0.012 (0.037)	-7.47 (0.24) ***<br>0.034 (0.024)	-7.28 (0.10) ***<br>0.01 (0.01)

Table S6 | The DOM bulk metrics did not change over time. We tested if the average intensity-weighted relative bulk DOM metrics changed over time using linear models for each DOM source. Values show the mean (± standard error) for the intercept (top set of values in each cell) and effect of time (bottom set of values) with the percent change in the relative abundance between day 1 and day 14. Proportion of bioavailable formulae were logit transformed. Statistical significance is indicated with *** at $p < 0.001$, ** at $p < 0.01$ and * at $p < 0.05$. Bolded and shaded value indicate compound classes that changed significantly over time with a greater than 10% change between day 1 and day 14.

Compound Class	Beech Leaf Leachate	Boreal Lake	Subarctic Lake	Subarctic River	Subtropical River	Temperate Marsh	Temperate Pond
H/C	1.14 (0.001) ***<br>< 0.001 (< 0.001)	1.16 (0.01) ***<br>< 0.001 (< 0.001)	1.16 (0.002) ***<br>< 0.001 (< 0.001)	1.22 (0.003)***<br>< 0.001 (< 0.001)	1.06 (0.009)***<br>< 0.001 (< 0.001)	1.08 (0.001)***<br>-0.002 (0.001)	1.16 (0.004)***<br>< 0.001 (< 0.001)
O/C	0.53 (0.004) ***<br>< 0.001 (< 0.001)	0.56 (0.005) ***<br>0.001 (0.001)	0.56 (0.002)***<br>< 0.001 (< 0.001)	0.54 (0.001)***<br>-0.0004 (0.0001)**<br>-1.2%	0.58 (0.002)***<br>< 0.001 (< 0.001)	0.59 (<0.001)***<br>-0.0004 (0.0001)**<br>-0.85%	0.57 (0.002)***<br>< 0.001 (< 0.001)
m/z	286.83 (1.83) ***<br>0.06 (0.17)	331.35 (1.87)***<br>-0.35 (0.18)	340.03 (0.84)***<br>0.14 (0.08)	340.40 (0.79) ***<br>-0.08 (0.07)	367.66 (2.05)***<br>-0.18 (0.19)	349.36 (3.45)***<br>0.18 (0.34)	347.80 (0.88)***<br>-0.09 (0.09)
Proportion bioavailable	-1.40 (0.03) ***<br>-0.001 (0.003)	-2.43 (0.023) ***<br>0.002 (0.002)	-2.94 (0.008)***<br>< 0.001 (< 0.001)	-2.50 (0.013) ***<br>0.002 (0.001)	**-3.58 (0.02) *****<br>**-0.008 (0.002)****<br>**-10.4%**	-2.97 (0.04)***<br>-0.004 (0.004)	-2.65 (0.03)***<br>-0.001 (0.003)

Table S7 | Estimated 16S rRNA copy numbers and cell sizes of bacterial strains

Species	Closest related species based on whole genome sequence similarity	Estimated number of 16S copies[1] (from the rrnDB)	Estimated cell size
Arthrobacter sp.	*Paenarthrobacter aurescens*	5-6	NA[2]
Pantoea sp.	*Pantoea agglomerans*	7	0.5-1.0 by 1.0-3.0 µm [12]
Pseudomonas fluorescens		5 (from the complete genome sequence)	0.5-0.8 by 1.5-3.0 µm [13]
Pseudomonas fragi		8	0.5-0.8 by 1.5-3.0 µm [13][3]
Raoultella sp.	*Raoultella terrigena*	8	Cell length 1.60 ± 0.08 µm [14]
Sphingobacterium sp.	*Sphingobacterium multivorum*	7	NA[2]

[1]Estimated from the rrnDB database [15] for the closest related species based on whole genome sequence similarity
[2]Cell size information unavailable
[3]Estimated cell size for *Pseudomonas fluorescens*

Table S8 | List of contaminant formulae removed from the mass spectra

Identified contaminants		Known contaminants	
Formula	*Mass*	*Formula*	*Mass*
C6H10O4	146.057881	C6H12O5	163.061197
C7H4O6	184.000698	C9H18O3	173.11832
C7H6O3	138.031468	C11H14O3	193.087018
C8H4O9	243.985744	C14H22O	205.159789
C8H6O6	198.016457	C8H16O6	207.087412
C8H6O7	214.011704	C11H22O4	217.144533
C8H8O4S	200.014564	C14H22O2	221.154703
C9H10O5S	230.024658	C13H26O3	229.180918
C9H6O7	226.011429	C10H12N2O5	239.067345
C9H8O4S	212.014625	C14H21NO3	250.144867
C12H14O8	286.069000	C10H20O7	251.113627
C13H14O8	298.069353	C16H32O2	255.232954
C13H21N3O3	267.158337	C17H22O2	257.154703
C14H18O7	298.105299	C15H16O4	259.097583
C15H8O14	411.990662	C17H24O3	275.165268
C16H16O10	368.073681	C18H36O2	283.264254
C17H16O11	396.068756	C16H18O5	289.108147
C17H18O11	398.084733	C17H26O4	293.175833
C17H20O11	400.100517	C12H24O8	295.139841
C19H22O10	410.120629	C16H26O3S	297.152989
C19H24O10	412.136351	C17H28O3S	311.168639
		C18H22O5	317.139447
		C18H30O3S	325.184289
		C14H28O9	339.166056
		C19H32O3S	339.199939
		C23H32O3	355.227868
		C16H32O10	383.192271
		C20H18O9	401.087806
		C18H36O11	427.218486
		C27H30O15	593.151194
		C27H30O16	609.146108
		C33H40O20	755.204017

Supplementary References

1. Scheuerl T, Hopkins M, Nowell RW, Rivett DW, Barraclough TG, Bell T. Bacterial adaptation is constrained in complex communities. *Nat Commun* 2020;**11**:754. https://doi.org/10.1038/s41467-020-14570-z
2. Fortmann-Grote C, Hugoson E, Summers J, Theodosiou L, Rainey PB. Genome Update for *Pseudomonas fluorescens* Isolate SBW25. *Microbiol Resour Announc* 2023;**12**:e00637-22. https://doi.org/10.1128/mra.00637-22
3. Pedrioli PGA, Eng JK, Hubley R, Vogelzang M, Deutsch EW, Raught B, et al. A common open representation of mass spectrometry data and its application to proteomics research. *Nat Biotechnol* 2004;**22**:1459–66. https://doi.org/10.1038/nbt1031
4. Gatto L, Lilley KS. Msnbase-an R/Bioconductor package for isobaric tagged mass spectrometry data visualization, processing and quantitation. *Bioinformatics* 2012;**28**:288–9. https://doi.org/10.1093/bioinformatics/btr645
5. Schum SK, Brown LE, Mazzoleni LR. MFAssignR: Molecular formula assignment software for ultrahigh resolution mass spectrometry analysis of environmental complex mixtures. *Environ Res* 2020;**191**:110114. https://doi.org/10.1016/j.envres.2020.110114
6. Tolić N, Liu Y, Liyu A, Shen Y, Tfaily MM, Kujawinski EB, et al. Formularity: Software for Automated Formula Assignment of Natural and Other Organic Matter from Ultrahigh-Resolution Mass Spectra. *Anal Chem* 2017;**89**:12659–65. https://doi.org/10.1021/acs.analchem.7b03318
7. Merder J, Freund JA, Feudel U, Hansen CT, Hawkes JA, Jacob B, et al. ICBM-OCEAN: Processing Ultrahigh-Resolution Mass Spectrometry Data of Complex Molecular Mixtures. *Anal Chem* 2020;**92**:6832–8. https://doi.org/10.1021/acs.analchem.9b05659
8. Herzsprung P, Hertkorn N, Tumpling W Von, Harir M, Friese K, Schmitt-Kopplin P. Understanding molecular formula assignment of Fourier transform ion cyclotron resonance mass spectrometry data of natural organic matter from a chemical point of view. *Anal Bioanal Chem* 2014;**406**:7977–87. https://doi.org/10.1007/s00216-014-8249-y
9. Koenker R. quantreg: Quantile Regression. 2023. https://doi.org/10.32614/CRAN.package.quantreg
10. Hawkes JA, D'Andrilli J, Agar JN, Barrow MP, Berg SM, Catalán N, et al. An international laboratory comparison of dissolved organic matter composition by high resolution mass spectrometry: Are we getting the same answer? *Limnol Oceanogr Methods* 2020;**18**:235–58. https://doi.org/10.1002/lom3.10364
11. Sofo A, Ricciuti P. A Standardized Method for Estimating the Functional Diversity of Soil Bacterial Community by Biolog® EcoPlatesTM Assay—The Case Study of a Sustainable Olive Orchard. *Applied Sciences* 2019;**9**:4035.
12. Manulis S, Barash I. Pantoea agglomerans pvs. gypsophilae and betae, recently evolved pathogens? *Mol Plant Pathol* 2003;**4**:307–14. https://doi.org/10.1046/j.1364-3703.2003.00178.x
13. Schober I, Koblitz J, Sardà Carbasse J, Ebeling C, Schmidt ML, Podstawka A, et al. BacDive in 2025: the core database for prokaryotic strain data. *Nucleic Acids Res* 2025;**53**:D748–D756. https://doi.org/10.1093/nar/gkae959
14. Tantasuttikul A, Mahakarnchanakul W. Growth parameters and sanitizer resistance of Raoultella ornithinolytica and Raoultella terrigena isolated from seafood processing

plant. *Cogent Food Agric* 2019;**5**:1569830. https://doi.org/10.1080/23311932.2019.1569830

15. Stoddard SF, Smith BJ, Hein R, Roller BRK, Schmidt TM. rrnDB: improved tools for interpreting rRNA gene abundance in bacteria and archaea and a new foundation for future development. *Nucleic Acids Res* 2015;**43**:D593–D598. https://doi.org/10.1093/nar/gku1201
